# The near-complete genome assembly of *Ampelopsis grossedentata* provides insights into its origin, evolution, and the regulation of flavonoid biosynthesis

**DOI:** 10.3389/fpls.2025.1580779

**Published:** 2025-08-11

**Authors:** Zhi Yao, Zhi Feng, Fuwen Wu, Peiling Zhang, Qiye Wang, Binling Ai, Yiqiang Wang, Meng Li

**Affiliations:** ^1^ Key Laboratory of Forestry Biotechnology of Hunan Province, Central South University of Forestry and Technology, Changsha, China; ^2^ Uelushan Laboratory Carbon Sinks Forests Variety Innovation Center, Central South University of Forestry and Technology, Changsha, China; ^3^ College of Biological, Hunan Normal University, Changsha, China; ^4^ Institute of Tropical Bioscience and Biotechnology, Chinese Academy of Tropical Agricultural Sciences, Haikou, Hainan, China

**Keywords:** *Ampelopsis grossedentata*, reference genome, WGD, WGCNA, AgF3H1

## Abstract

*Ampelopsis grossedentata*, native to southern China, is renowned for its therapeutic and nutritional benefits, often called the “king of flavonoids” due to its high dihydromyricetin content. The dried stems, leaves, and shoot tips, known as “vine tea,” are consumed as a health beverage and traditional remedy for colds and fever. In this study, we assembled a near-complete reference genome of *A. grossedentata* spanning 555.42 Mb, where Hi-C-based correction resolved 18 out of its 20 chromosomes into gap-free assemblies. The genome, anchored to 20 chromosomes, comprises 44 contigs with an N50 of 21.93 Mb and 28 scaffolds with an N50 of 30.45 Mb, containing 25,999 protein-coding genes and 62.62% repetitive sequences. The *A. grossedentata* experienced two whole-genome duplication (WGD) events: a whole-genome triplication event shared by the core angiosperms and a WGD event shared with Vitaceae family. Through transcriptome-metabolome integrated analysis, *AgF3H1* gene was identified as playing a crucial role in the biosynthesis of dihydromyricetin (a flavanonol) in *A. grossedentata*. The *AgF3H* gene is essential for converting pentahydroxy flavones to dihydromyricetin within the flavonoid biosynthesis pathway in *A. grossedentata*, as confirmed by molecular docking results. Thus, we postulate that *AgF3H1* serves as a pivotal regulatory gene in the dihydromyricetin biosynthetic pathway of *A. grossedentata*. These insights offer valuable genetic resources for the molecular breeding of *A. grossedentata* and enhance our comprehension of Vitaceae genomic evolution and flavonoid biosynthesis regulation in medicinal and nutritional plants.

## 1 Introduction

*Ampelopsis grossedentata*, a unique Vitaceae family in China, is an ancient medicinal and edible plant mainly found in southern Yangtze River such as Guangdong, Hunan and Hubei (Gu et al., 2020; Cao et al., 2023). According to the “National Compilation of Chinese Herbal Medicines,” the whole plant is used for clearing heat, detoxifying, lowering liver and lowering blood pressure to treat cold, fever and hepatitis (Ma et al., 2019; Li et al., 2022). Using young stems and leaves, it is processed by enzyme inactivation, rolling and drying to make “vine tea,” also known as “Maoyan terry tea,” which is helpful to relieve cough, eliminate phlegm and dispel wind and dampness (Carneiro et al., 2021; Luo et al., 2023). Vine tea was first noted in the “Classic of Tea” and was traditionally consumed by Zhuang and Yao ethnic minorities before gaining popularity among other minorities like the Tujia, Dong, and Hakka (Wu et al., 2023).

Flavonoids are secondary metabolites widely existing in plants and play an important role in plant defense and development, while also possessing significant health and medicinal values, making them a focus of attention (Shen et al., 2022; Liu et al., 2023). Contemporary pharmacological research indicates that *A. grossedentata*’s tender stems, leaves, and shoot tips predominantly contain flavonoids (Zeng et al., 2023). With its total flavonoid content reaching 35%-45%, it is the plant with the highest known concentration of flavonoids, providing it significant commercial potential and vast market opportunities (Zhang et al., 2016, Zhang X. et al., 2019). Dihydromyricetin is a naturally occurring dihydroflavonol compound, primarily derived from *A. grossedentata* (Zeng et al., 2023). In the extract of *A. grossedentata*, dihydromyricetin accounts for about 35% of the total flavonoids, making them the most abundant flavonoid monomer compounds in the plant (Zhang et al., 2018; Hu et al., 2020). Dihydromyricetin has significant pharmacological effects such as antihyperglycemic (Chen et al., 2016; Ran et al., 2019), antioxidation (Ye et al., 2015; Xie et al., 2019), anti-tumor (Zhou et al., 2014; Guo et al., 2019), anti-inflammatory (Hou et al., 2015; Zhang et al., 2022), and antibacterial properties (Wu et al., 2017; Xiong et al., 2021). Therefore, *A. grossedentata* is commonly used as dietary supplements such as teas, beverages, and lozenges (Carneiro et al., 2020; Zhang et al., 2021). Additionally, *A. grossedentata* extract can inhibit melanin production and is widely used in skin-whitening products (Huang et al., 2016). Enhancing the flavonoid content in *A. grossedentata* is crucial for augmenting its medicinal and nutritional benefits.

The flavonoid production process involves anthocyanin, isoflavonoid, flavone, and flavonol pathways, initiated by enzymes chalcone synthase, chalcone isomerase, and flavanone 3-hydroxylase (F3H) (Zeng et al., 2013; Ni et al., 2020). The *F3H* gene encodes a key enzyme for flavonol biosynthesis, which catalyzes the conversion of flavanones into dihydrokaempferol, dihydroquercetin, and dihydromyricetin (Prescott and John, 1996). To date, *F3H* genes have been cloned from various plant species, including *Malus pumila* (Davies, 1993), *Vitis vinifera* (Sparvoli et al., 1994), *Arabidopsis thaliana* (Pelletier and Shirley, 1996), and *Glycine max* (Zabala and Vodkin, 2005). Current research on *A. grossedentata* primarily focuses on its pharmacological effects, antioxidant activity, physiological and biochemical characteristics, transcriptome sequencing, and chloroplast genome analysis (Ye et al., 2015; Huang et al., 2016; Gu et al., 2020; Luo et al., 2023; Wu et al., 2023). Based on transcriptome sequencing, Li et al. (2020) and Yu et al. (2021) predicted key genes involved in the flavonoid and dihydromyricetin biosynthetic pathways in *A. grossedentata*, respectively. Zhang et al. (2022), discovered an *AgF3H* gene through RNA-seq, showed the full-length CDS from leaf cDNA, and confirmed its expression in *Saccharomyces cerevisiae*, providing insights into dihydromyricetin hydroxylation in *A. grossedentata*. Although multiple genes involved in flavonoid biosynthesis have been identified in *A. grossedentata*, the accumulation patterns of flavonoids in different tissues and molecular regulatory mechanisms remain unclear.

**Figure 1:**
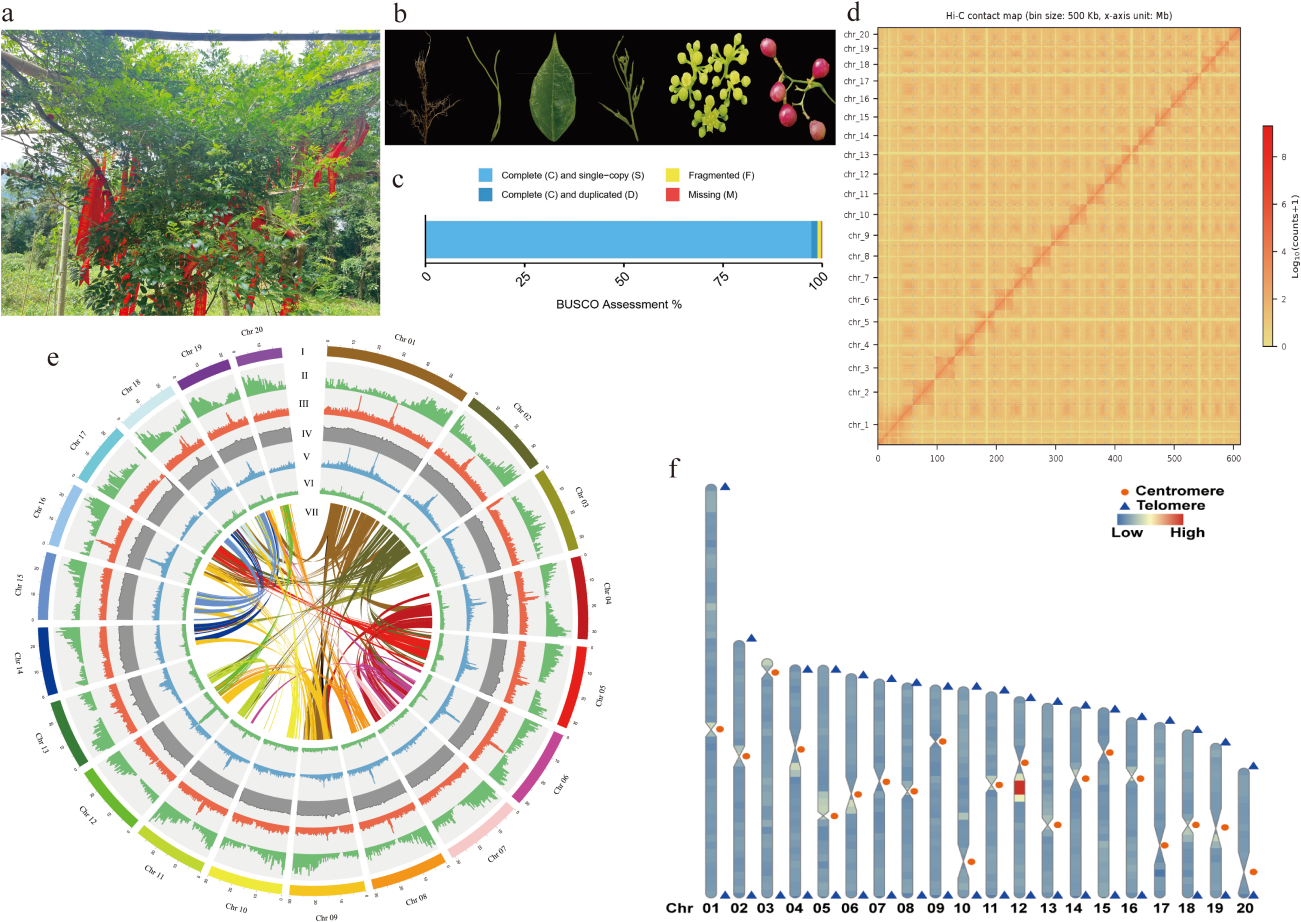
Comprehensive genome assembly of *A. grossedentata*. **(a)** Botanical overview of *A. grossedentata*. The red element is the silk used by local residents for blessings. **(b)** Microscopic images of *A. grossedentata* tissues. From left to right: root, stem, leaf, shoot tip, flower, and fruit. **(c)** BUSCO analysis of the *A. grossedentata genome*. **(d)** Hi-C heatmap of the assembled genome. Horizontal coordinate: Length of the chromosome; Vertical coordinate: The name of the chromosome; Legend: The frequency of interaction. The size of bin is 500kb. **(e)** Chromosomal features: (I) chromosome length; (II) gene density; (III) TE density; (IV) GC content; (V) LTR density; (VI) repeat coverage; (VII) synteny. All features are displayed at 500 kb resolution. **(f)** Telomere Identification: Triangles and circles denote telomeres and centromeres, with warm and cool tones indicating regions of high and low TE density, respectively.

In this study, we utilized wild *A. grossedentata* samples from Yongshun County, Hunan Province (PZY009, **Figure 1a**). Using Illumina, PacBio, and ONT ultra-long reads combined with Hi-C technology, we constructed the first near-complete reference genome for *A. grossedentata*. We performed transcriptomic and metabolomic analyses on the roots, shoot tips, stems, and leaves of PZY009 (**Figure 1b**) at the same developmental stage to identify key enzyme genes involved in the flavonoid biosynthesis pathway. This research provides valuable insights into the evolution, molecular-assisted breeding, and chemical diversity of *A. grossedentata*’s bioactive compounds.

## 2 Materials and methods

### 2.1 Plant materials and genome sequencing

The roots, stems, leaves, and shoot tips of wild germplasm (PZY009) (**Figure 1b**) were collected from the *A. grossedentata* garden in Yongshun County, Xiangxi Autonomous Prefecture, Hunan Province (29°16′42″N, 109°53′17″E). Samples were flash-frozen with liquid nitrogen and stored at -80°C.

High-quality genomic DNA was extracted from *A. grossedentata* leaves using a modified CTAB method (Allen et al., 2006). DNA integrity and purity were verified by agarose gel electrophoresis and spectrophotometer. Illumina short-read genome libraries (2×150 bp) were prepared according to Illumina standard protocols and sequenced on the Illumina NovaSeq platform. ONT ultra-long read sequencing was performed on the PromethION sequencer. PacBio sequencing included generating long-read libraries from genomic DNA fragments up to 15kb, which were sequenced on the PacBio Sequel II platform to produce HiFi (CCS) reads. Hi-C libraries were constructed using the HindIII restriction enzyme and sequenced on the Illumina NovaSeq chromosome assembly platform.

For comprehensive transcriptome analysis, RNA was isolated from roots, shoot tips, stems, and leaves using a plant RNA extraction kit according to the standard protocol. Following Oxford Nanopore Technologies’ strand-switching protocol, mRNA was enriched to synthesize cDNA. PCR-amplified cDNA was sequenced using the PromethION sequencer.

### 2.2 Genome assembly and assessment

We evaluated genome size, heterozygosity, and repeat content using 17-mer k-mer distribution from Illumina short reads. Raw PacBio subreads were filtered and corrected via the PacBio circular consensus sequencing (pbccs) pipeline (https://github.com/PacificBiosciences/ccs) and assembled *de novo* using hifiasm software (v0.16.1-r375) (Cheng et al., 2021). Pilon software was used for primary contig correction. Genome assembly quality was assessed using BWA-MEM (v0.7.17) (Li and Durbin, 2009), CEGMA (Parra et al., 2007), and BUSCO (v5.2.2) (Simão et al., 2015). Hi-C chromosome conformations were captured using a DNase-based method and sequenced in 150 bp paired-end mode on Illumina NovaSeq (Ramani et al., 2020).

When processing Illumina DNA sequencing data, we utilized fastp (v0.21.0) software to perform filtering, removing low-quality reads and adapter sequences (Chen et al., 2018). Then, we utilized *K*-mer-based analysis methods (Marçais and Kingsford, 2011) using Jellyfish (v2.2.7, https://academic.oup.com/bioinformatics/article/27/6/764/234905?login=true) and Genome Characteristics Estimation (GCE) software (Liu et al., 2013) to estimate genome size and heterozygosity rate. To confirm whether the sequencing data contained contamination, we used blast+ (Camacho et al., 2009) to extract the first 50,000 reads and compared them with the NT nucleotide sequence database. Finally, we used MEGAN (Huson et al., 2016) for species classification.

We used Filtlong (v 0.2.1, https://biocontainers.pro/tools/filtlong) and Porechop (v0.2.4, https://github.com/rrwick/Porechop) to remove short reads (<10 kb) and adapter sequences, then used NextDenovo for preliminary assembly of ONT ultra-long reads. Subsequently, we corrected the ONT draft genome using Racon (https://github.com/isovic/racon) and ONT ultra-long sequencing data, as well as Pilon (v1.24, Release Pilon version 1.24 · broadinstitute/pilon) and Illumina sequencing data. For the PacBio HiFi draft genome assembly, we used CCS reads to filter out low-quality sequences, then assembled the genome with hifiasm (v0.16.1-r375).

We adopted Purge_dups software (https://link.zhihu.com/?target=https%3A//github.com/dfguan/purge_dups) (Guan et al., 2020) to clear haplotypes, and used minimap2 (v2.28) (Li, 2018) for mitochondrial and chloroplast sequence alignment, filtering out sequences with over 50% base pair alignment. Furthermore, we eliminated bacterial contamination using the BLAST refseq database, while also removing poorly supported contigs (McGinnis and Madden, 2004). In this step, the use of fastp (Chen et al., 2018) allowed us to filter the raw Hi-C sequencing data to obtain purer data. Then, HICUP (Wingett et al., 2015) mapped the clean data to the genome assembly, thereby removing unmapped reads, invalid pairs, and duplicates.

In the process of generating the genome draft, we used ALLHiC software (v 0.9.8) (Zhang X. T. et al., 2019) and successfully generated a 2n karyotype genome draft through agglomerative hierarchical clustering. Additionally, by utilizing 3D-DNA (Dudchenko et al., 2017) and Juicer (v1.5) (Durand et al., 2016b), we converted the interactions of contigs into specific binary files. This process was visualized using Juicebox (Durand et al., 2016a), guiding the manual ordering and orientation of contigs. Based on this, we manually removed redundant contigs according to interaction relationships, while filling gaps with 100 Ns. We also used HiCExplorer (Wolff et al., 2020) to plot the interaction strength and positional relationship of contigs.

### 2.3 Identification of telomeres and centromeres

To achieve reference genome assembly, we used winnowmap (v1.11) (Jain et al., 2020) with parameters k=15 and –MD to align the fill-in data with the genome gap regions, in order to fill the gaps in the genome. If the alignment spans both ends of a gap, we select the longest and best alignment to replace the gap region. Next, we used Winnowmap2 (parameters: k=15, dissimilarity > 0.9998, -MD, ax map-pb) (Jain et al., 2022) to align the revised genome containing filled gaps with HiFi reads ≥10 kbp in size. Telomere repeat sequences (AAACCCT at the 5’ and 3’ ends) of all reads were searched, with the most abundant reads marked as reference and the rest as queries. Then, we used medaka_consensus to assemble these reference and query sequences. Subsequently, we used nucmer (v3.1) (Kurtz et al., 2004) to replace the terminal sequences on each pseudo-chromosome. Finally, we used Racon and Pacbio HiFi reads to perform error correction on the near-complete reference genome assembly. Centromere and telomere identification were carried out using CentIER (v3.0) (Xu et al., 2024) and A Telomere ldentification toolKit (tidk, v0.2.63) (Brown et al., 2025) with default parameters, respectively.

### 2.4 Genome annotation

We initially annotated tandem repeat sequences using Genome-wide Microsatellite Analyzing Toward Application (GMATA, v21, https://github.com/XuewenWangUGA/GMATA.git) and Tandem Repeats Finder (v4.10, TRF) (Benson, 1999). We integrated ab initio and homology-based methods to annotate transposable elements (TEs) within the *A. grossedentata* genome. Specifically, we employed MITE-hunter (Han and Wessler, 2010) and RepeatModeler2 (v1.0.11) (Flynn et al., 2020) with default parameters to predict the ab initio repeat library of *A. grossedentata*. We then developed an LTR-RT library using LTRharvest (Ellinghaus et al., 2008) and LTR_Finder (Ou and Jiang, 2019) with default settings, and created a non-redundant LTR-RT library through LTR_retriever (Ou and Jiang, 2018). These libraries were compared with the TEclass repbase (v20170127; https://www.girinst.org/) (Zhuo and Feschotte, 2015) database to classify the repeat families. Finally, the LTR_retriever, MITE-Hunter, and RepeatModeler2 (v1.0.11) libraries were merged and input into RepeatMasker (v4.0.7) (Chen, 2004) to annotate repetitive elements in each assembled genome. LTR, Copia, and Gypsy insertion times were estimated using LTR_retriever with default parameters.

Gene structure was annotated using homology search, *de novo* prediction, and reference-guided transcriptome assembly. In the homology prediction process, we used blast+ (Camacho et al., 2009) to locate protein sequences on the reference genome, and then used Exonerate to predict transcripts and coding regions (Slater and Birney, 2005). Additionally, genes predicted by BUSCO were incorporated into the homology prediction results, which was done during genome quality assessment (Manni et al., 2021). For *de novo* gene prediction, we relied on Augustus (v3.3) (Stanke et al., 2008) and GlimmerHMM (Delcher et al., 2007) with default parameters, operating through training sets. For RNA-seq reads, we used fastp (Chen et al., 2018) for filtering and HISAT2 (Kim et al., 2019) for genome alignment. The alignment results were then used as input for Stringtie to obtain transcripts (Kovaka et al., 2019), followed by prediction using TransDecoder (https://github.com/TransDecoder/TransDecoder). For Nanopore RNA-seq reads, we used NanoFilt (v2.8.0, https://github.com/wdecoster/nanofilt) for filtering and then Pychopper (https://github.com/epi2me-labs/pychopper) to determine full-length sequences. Post error correction using racon, these full-length sequences were aligned to the genome using minimap (Li, 2016).

The alignment results were fed into Stringtie to obtain transcripts (Kovaka et al., 2019). Finally, all predicted gene sets were combined into one gene set through MAKER (Holt and Yandell, 2011) and further optimized to obtain the final gene set. Lastly, we used BUSCO (Simão et al., 2015) to verify the completeness of the genome annotation to ensure the reliability and accuracy of our work.

Protein functions were predicted by comparing protein sequences against multiple public databases using DIAMOND (Buchfink et al., 2015). Databases utilized included non-redundant database (Deng et al., 2006), Swiss-Prot (Boeckmann et al., 2003), eggNOG (http://eggnog5.embl.de/), Gene Ontology (GO, https://www.geneontology.org/), and Kyoto Encyclopedia of Genes and Genomes (KEGG, https://www.genome.jp/kegg/) (Kanehisa and Goto, 2000). This comparative analysis aimed to identify associated gene functions, conserved motifs, and protein domains. Annotation was performed via KOBAS (Xie et al., 2011), and putative domains and GO terms of genes were identified using InterProScan with default settings (Blum et al., 2021). BLAST+ (Camacho et al., 2009) was used to compare the EvidenceModeler-integrated (Haas et al., 2008) protein sequences against the four major public protein databases with an E value cutoff of 1e−05, retaining results with the lowest E value.

Non-coding RNAs (ncRNAs) were classified into categories such as miRNAs, rRNAs, tRNAs, snoRNAs, and snRNAs. To identify ncRNAs, we employed two strategies: database searching and model prediction. tRNAs were predicted using tRNAscan-SE with eukaryote parameters (Chan et al., 2021). MicroRNAs, rRNAs, snRNAs, and snoRNAs were detected using Infernal cmscan (Nawrocki and Eddy, 2013) against the Rfam database. (https://rfam.xfam.org/). rRNAs and their subunits were predicted using RNAmmer (Lagesen et al., 2007).

### 2.5 Comparative genomic analysis

In comparative genomic studies, to ensure research accuracy, we selected species with high-quality genome assemblies that are phylogenetically closely related to the target species (*A. grossedentata*). Accordingly, three *Vitis* species (*V. vinifera*, *V. rotundifolia*, and *V. amurensis*) along with *Cissus rotundifolia* from the Vitaceae family were prioritized. Additionally, we included model plants (*Arabidopsis thaliana* and *Oryza sativa*) and multiple species with elevated flavonoid contents: *Glycine max*, *Papaver somniferum*, *Salvia miltiorrhiza*, *Solanum lycopersicum*, *Dendrobium officinale*, and *Scutellaria baicalensis*. *Nymphaea colorata* was designated as the outgroup based on its phylogenetic distance as a monocotyledonous plant from *A. grossedentata*, a eudicot species (**Supplementary Table S13**). Genetic family clustering of these 14 plant species was conducted using BLAST+ (Camacho et al., 2009) and OrthoFinder (Emms and Kelly, 2019). Gene families were annotated using the Panther database (Mi et al., 2019). Unique gene families for each species were identified through GO and KEGG enrichment analysis, facilitated by clusterProfiler (Yu et al., 2012). Single-copy orthologous genes were extracted and aligned using MUSCLE (Edgar, 2004), with alignment results filtered by TrimAl (Capella-Gutiérrez et al., 2009) and consolidated into a supermatrix alignment.

A Maximum-likelihood (ML) phylogenetic tree was constructed via RAxML employing the PROTGAMMAWAG model (Stamatakis, 2014). Divergence times between species were estimated using the MCMCTree program in PAML (Yang, 2007), with burn-in=10,000, sample-number=100,000, and sample-frequency=2, using calibration times from the TimeTree database (Kumar et al., 2022). Including: *N. colorata* – *O. sativa*: 168–191 Mya; *D. officinale* – *O. sativa*: 108–123 Mya; *P. somniferum* – *O. sativa*: 142–163 Mya; *P. somniferum* – *A. thaliana*: 126–136 Mya; *V. vinifera* – *A. thaliana*: 109–124 Mya; *G. max* – *A. thaliana*: 102–112 Mya; *S. lycopersicum* – *A. thaliana*: 111–123 Mya; *S. lycopersicum* – *S. miltiorrhiza*: 75–96 Mya; *S. baicalensis* – *S. miltiorrhiza*: 33–72 Mya; *V. vinifera* – *C. rotundifolia*: 31–96 Mya; *V. vinifera* – *V. rotundifolia*: 4–14 Mya; *V. vinifera* – *V. amurensis*: 5–40 Mya; *V. vinifera* – *C. rotundifolia*: 31–96 Mya. In brief, an all-againstall BlastP search was performed on the 14 proteomes using DIAMOND (Buchfink et al., 2015) with a cutoff e-value of 10-5. HOGs were obtained using PhyloMCL (Zhou et al., 2020) with default parameters. For each HOG, PASTA (Tang and Riva, 2013) was used for multiple sequence alignment, and protein alignments were converted to nucleotide alignments. A maximum likelihood tree was reconstructed for each HOG using IQ-TREE2 (Minh et al., 2020) with 100 bootstrap replicates. The GDs on each gene tree were estimated using a previously described strategy (Ren et al., 2018) if nodes on the GF tree had >50% bootstrap support. Patterns of duplicate retention for GD candidates were counted for further evaluation.

Gene family evolution was modeled as a random birth and death process, with expansion and contraction rates of one gene per million years. CAFE software (Han et al., 2013) was used to predict gene family changes in Ampelopsis cordata relative to its ancestors, with a p-value threshold of 0.05 to identify significant size changes. Phylogenetic tree topology and branch lengths informed the significance of these changes.

Single-copy orthologous genes were aligned via MUSCLE (Edgar, 2004), and positive selection was analyzed using PAML CodeML (Yang, 2007), considering *A. grossedentata* as the foreground branch. P-values were determined using χ2 statistics, with FDR correction for multiple testing.

Collinearity analysis involved using DIAMOND (Buchfink et al., 2015) to identify similar gene pairs between species (e < 1E-5, C-score > 0.5, filtered by JCVI software) (Tang et al., 2024). Adjacent similar gene pairs of chromosomes were determined based on the gff3 file, and collinear blocks were identified using MCScanX (parameters: -a -e 1e-5 -s 5) (Wang et al., 2012), with circular plots generated via the R package circlize (Gu et al., 2014). Syntenic blocks were identified using ‘jcvi.compara.catalog orthologs –cscore=0.7’ (Tang et al., 2024), and genes from all collinear blocks were obtained.

To identify whole-genome duplications (WGD) in the Ampelopsis cordata genome, we utilized a comprehensive WGD and intra-genome collinearity detection tool, along with Ks estimation and peak fitting (Sun et al., 2022). The combined use of 4DTv and Ks values of syntenic regions is a widely accepted method for detecting WGD events. For *A. grossedentata*, WGD events were identified using the WGD software (Zwaenepoel and Van de Peer, 2019).

### 2.6 Transcriptome analysis

Adapters were initially filtered from the raw RNA short-reads, followed by the removal of poly(A) tails and low-quality reads (Q < 20). The remaining high-quality reads were used to determine Q20, Q30, and GC content. These clean reads were then mapped to the reference genome and full-length transcript using HISAT (Kim et al., 2019). Reads with perfect matches or a single mismatch were utilized to reconstruct transcripts via StringTie (Kovaka et al., 2019). Expressed genes were identified based on mapping results. If reads aligned with annotated gene sequences, the gene was classified as existing and coded accordingly. Otherwise, if reads aligned with the full-length transcript but not with any annotated sequence, the gene was considered novel and recorded as a new identification.

Gene expression was quantified using fragments per kilobase of transcript per million mapped reads (FPKM). Read counts from the sequenced library were normalized using a scaling factor in edgeR (Robinson et al., 2010). Differential expression of dgps paralogs across roots, stems, leaves, and shoot tips was analyzed with EBSeq, while DESeq2 (Anders and Huber, 2010) was used for cross-tissue comparisons. Significant differential expression was determined with FDR < 0.05 and |log2(foldchange)| ≥ 2. GO and KEGG enrichment analyses of DEGs were performed via clusterProfiler (Wu et al., 2021). The PPI network was analyzed using NetworkAnalyst and STRING.

### 2.7 Widely targeted metabolomic analysis

Raw data were converted to mzXML format using MSConvert from the ProteoWizard software suite (Rasmussen et al., 2022) and processed in R with XCMS for feature detection (Navarro-Reig et al., 2015), retention time correction, and alignment. Metabolites were identified by matching accurate mass and MS/MS data with HMDB (Wishart et al., 2007), MassBank (Horai et al., 2010), Knapsack, ReSpect, LipidMaps (Sud et al., 2007), KEGG (https://www.genome.jp/kegg/), and a proprietary database from Panomix Biomedical Tech Co., Ltd. (Suzhou, China). Metabolite molecular weights were determined by the m/z ratios of parent ions. Molecular formulas were predicted using ppm and adduct ions and matched with databases for MS identification. MS/MS data was concurrently matched with fragment ions and database information for identification.

We employed two multivariate statistical analysis models, unsupervised (PCA) and supervised (PLS-DA, OPLS-DA), to differentiate groups using the R ropls package (Thévenot et al., 2015). Statistical significance was determined by P.value from group comparisons. Biomarker metabolites were filtered based on P-value, VIP (variable importance in projection from OPLS-DA), and fold change. Metabolites with P < 0.05 and VIP > 1 were deemed significantly differentially expressed.

Differential metabolites underwent pathway analysis via MetaboAnalyst (Xia and Wishart, 2011), integrating pathway enrichment and topology analyses. The identified metabolites were mapped to KEGG pathways for biological interpretation, and visualizations were created using the KEGG Mapper tool.

### 2.8 Weighted gene co-expression network analysis

For co-expression network analysis to detect high gene correlation modules, we used the WGCNA package in R (Zhang and Horvath, 2005). Modules associated with phenotypic traits were identified by converting the adjacency matrix to a topological overlap matrix and filtered using the WGCNA goodGenes function. The hierarchical gene clustering tree was pruned with the cutreeDynamic function, and modules with correlation coefficients (r) above 0.75 were merged. The gene co-expression network was constructed using the blockwiseModules function with an unsigned TOMType. Module eigengenes were computed via the WGCNA’s module eigengenes function, and their association with phenotypic traits was evaluated using Pearson correlation analysis. Hub genes were identified using the CytoHubba plugin (Chin et al., 2014) in Cytoscape (Shannon et al., 2003).

### 2.9 Molecular docking of *AgF3H* genes

AlphaFold3 (Abramson et al., 2024) predicted the crystal structure of the *AgF3H* protein in *A. grossedentata*. The crystal structure was processed using the Protein Preparation Wizard module in Schrödinger for preprocessing, native ligand state regeneration, H-bond optimization, energy minimization, and water removal (Abramson et al., 2024). The 2D sdf files of pentahydroxyflavanone, naringenin, and eriodictyol were converted into 3D chiral conformations using the LigPrep module in Schrödinger. The SiteMap module pinpointed the optimal binding site, while the Receptor Grid Generation module configured the most appropriate enclosing box for this site, thereby defining the active site of the *AgF3H* proteins. Pentahydroxyflavanone, naringenin, and eriodictyol were docked to the active sites of *AgF3H1* and *AgF3H2* proteins using high-precision XP docking. MM-GBSA calculations provided the binding free energy (dG Bind) between the ligands and proteins, where lower values indicate more stable binding.

Per the manufacturer’s guidelines, the M-MLV reverse transcriptase kit was utilized to synthesize the first-strand cDNA for qRT-PCR analysis. The qRT-PCR was conducted with the iTaq Universal SYBR Green super mix and recorded by the ABI 7500 PCR system. The procedure was replicated three times, with each iteration including standards and negative controls. The qRT-PCR protocol entailed a 30s denaturation at 95°C, followed by 40 cycles of 5s denaturation at 95°C, 30s annealing at 60°C, and a 20s extension at 60°C. Each sample was run thrice, and the qRT-PCR outcome was averaged from three replicate applications. The standard gene GAPDH of *A. grossedentata* served as the internal reference gene (Xu, 2017), with Ct values determining the relative expression of *AgF3H1* (Livak and Schmittgen, 2001) (**Supplementary Table S21**).

## 3 Results

### 3.1 Sequencing and assembly of the *A. grossedentata* genome

We generated 70.8 Gb of high-quality paired-end reads on the Illumina platform for *k*-mer (k=17) analysis to estimate the genome size of *A. grossedentata* (**Table 1**; **Supplementary Figure S1**; **Supplementary Table S1**). The final assembled genome size of *A. grossedentata* was 555.42 Mb, with a GC content of 31.98%, a repeat sequence proportion of 62.62%, and a heterozygosity rate of 1.48% (**Table 1** and **Supplementary Table S1**), indicating a highly heterozygous and repetitive genome.

**Table 1:** Genome assembly and annotation statistics of *A. grossedentata*.

Genome information	Value
Revised genome size (Mbp)	555.42
Chromosome number	20
Contig number	44
Scaffold number	28
Contig N50 (bp)	21,931,686
Scaffold N50 (bp)	30,449,182
GC content (%)	31.98%
Heterozygous ratio (%)	1.48%
Repeats (%)	62.62%
*K-mer*	17
Number of genes	26,359
Number of miRNAs	122
Number of rRNAs	849
Number of tRNAs	497
Number of snoRNAs	609
Complete BUSCOs (%)	98.8%

We evaluated the genome assembly quality using sequence consistency and BUSCO metrics. Sequence consistency revealed a 99.02% alignment rate and 99.29% coverage rate of short reads to the *A. grossedentata* genome, indicating high consistency (**Supplementary Table S2**). BUSCO analysis showed 98.8% completeness for the 425 single-copy orthologous genes, confirming the high integrity of the assembly (**Figure 1c**).

The proportions of A, T, G, and C in the *A. grossedentata* genome were within normal ranges, with an N content of 0.00%, which is within the acceptable range (<10%) (**Supplementary Table S3**). The heterozygous SNP proportion was 0.2953%, and the homozygous SNP proportion was 6.7836e-05% (**Supplementary Table S4**), indicating high single-base accuracy. These results demonstrate that the *A. grossedentata* genome sequence has high consistency, accuracy, and completeness.

### 3.2 Hi-C technology assisted the assembly of near-complete reference genome of *A. grossedentata*

To achieve chromosome-level assembly, we utilized high-throughput chromosome conformation capture (Hi-C) sequencing technology, generating 69.8 Gb of 235.36 million paired-end Hi-C reads (**Supplementary Table S1**). Using Allhic software, we anchored 28 scaffolds, totaling 608.41 Mb of sequences, to the *A. grossedentata* genome (**Supplementary Table S5**). With the error correction and assembly assistance of Hi-C technology, we obtained 20 chromosome-level sequences, achieving a genome anchoring rate of 99.89% (**Supplementary Table S6**). Each chromosome contains at least one scaffold, with lengths ranging from 18.57 Mb (Chr 20) to 59.11 Mb (Chr 1) (**Supplementary Table S7**). The Hi-C interaction matrix heatmap demonstrated higher interaction intensity among adjacent sequences, with 20 pseudochromosomes aligned along the diagonal (**Figure 1d**). A circos map was drawn based on grape genome data (**Figure 1e**). Using 7-base telomere repeat sequences (AAACCCT) as queries, we identified 38 telomeres on 20 pseudochromosomes (except Chr 03 and Chr 17, each missing one telomere) and located potential centromeres on each chromosome. Detailed regions are listed in **Supplementary Tables S8 and S9**. The assembly is deemed a high-quality, near-complete genome (**Figure 1f**).

### 3.3 Repeat sequence prediction and genome annotation

Eukaryotic genomes’ repeat sequences play a crucial role in evolution, inheritance, and life variation, making them vital for comprehensive analysis of gene expression control, genome structure, and species evolution. Using homology, *de novo*, and transcriptome predictions, we foresaw a total of 25,756 genes in *A. grossedentata*, exceeding *N. colorata* (19,299), but trailing *V. vinifera* (29,591), *V. rotundifolia* (26,742), and *V. amurensis* (29,168) (**Supplementary Figure S2a**). The average gene length in *A. grossedentata* is 7895 bp, with an average exon length of 351 bp, an average intron length of 1241 bp, and an average coding region length of 1615 bp (**Supplementary Table S10**). Furthermore, 9848 genes (37.36%) and 25,990 genes (98.60%) had homologous gene predictions in the eggNOG and NR databases, respectively (**Supplementary Table S11**; **Supplementary Figure S2b**). Additionally, we identified 2,077 non-coding RNAs in the *A. grossedentata* genome, including 849 rRNAs, 497 tRNAs, 122 miRNAs, and 609 snoRNAs (**Supplementary Table S12**).

### 3.4 Comparative genomic analysis

**Figure 2:**
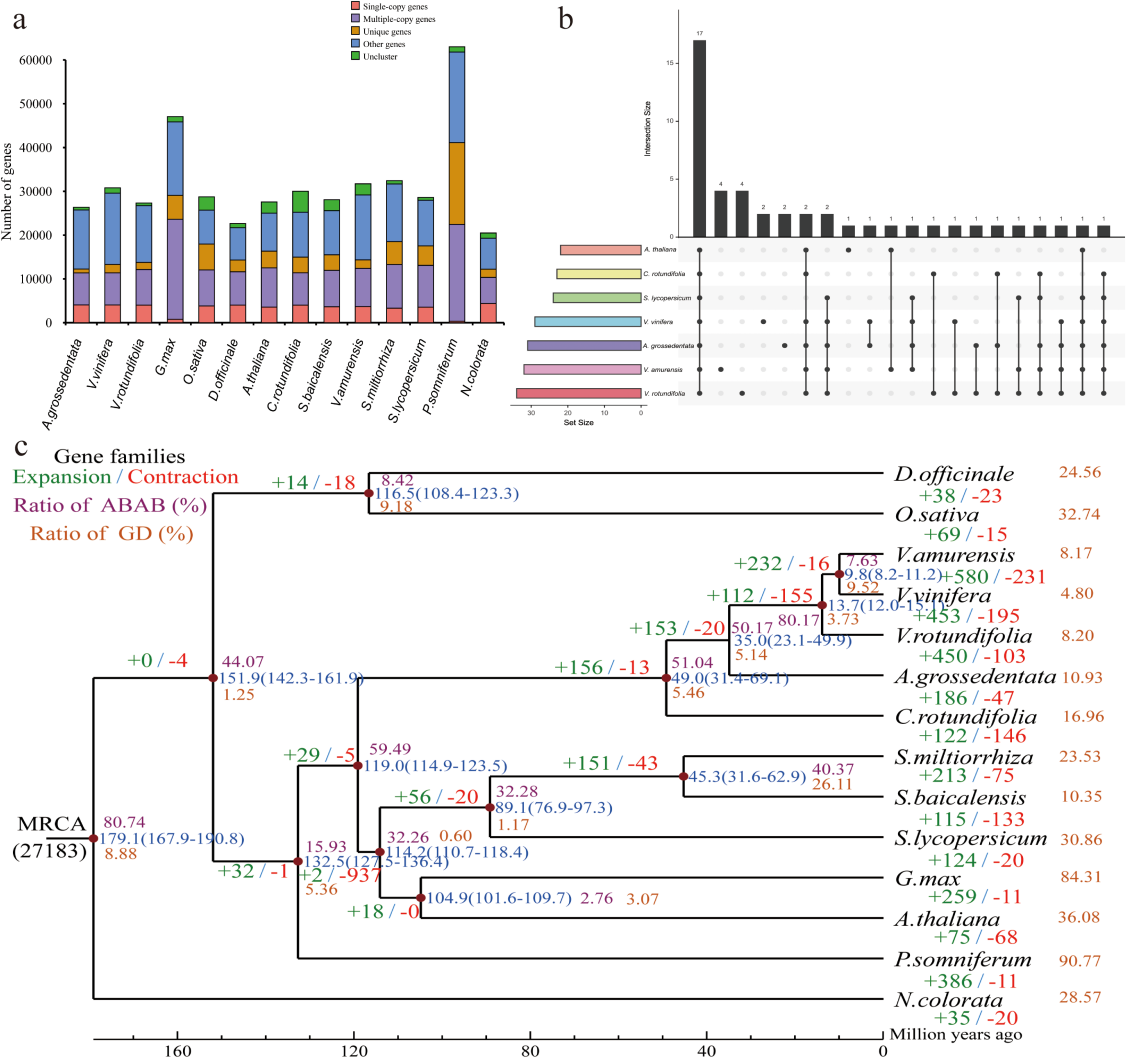
Gene family cluster and evolutionary analysis. **(a)** Gene family copy number across 14 species (V. vinifera, *V. rotundifolia*, *V. amurensis*, *C. rotundifolia*, *A. thaliana*, *G. max*, *D. officinale*, *P. somniferum*, *S. miltiorrhiza*, *S. lycopersicum*, *O. sativa*, *S. baicalensis*, *N. colorata* and *A. grossedentata*). **(b)** Upset diagram of shared and unique gene families among *A. grossedentata*, *V.vinifera*, *V.amurensis*, *V.rotundifolia*, *A. thaliana*, *C.rotundifolia* and *S. lycopersicum*. **(c)** Phylogenetic tree with divergence times and gene family expansion/contraction in 14 species. GD events in different eudicot lineages identified by reconciliation of gene trees and the species tree. Node numbers indicate estimated divergence times (Mya) with error ranges in blue parentheses; red and green numbers denote contracted and expanded gene families, respectively.

We analyzed the genes in the *A. grossedentata* genome against 12 other species (*V. vinifera, V. rotundifolia, V. amurensis, C. rotundifolia, A. thaliana, G. max, D. officinale, P. somniferum, S. miltiorrhiza, S. lycopersicum, O. sativa*, and *S. baicalensis*) and one outgroup species (*N. colorata*) (**Supplementary Table S13**). We utilized the genomes of these species for homologous gene identification, gene family clustering, and analysis of single-copy gene enrichment in *A. grossedentata* (**Figure 2a**). The analysis revealed 27,473 gene families across the 14 species, with 6,738 gene families being conserved, including 175 single-copy gene families shared among all species (**Figure 2a**). We extracted clustering data for *C. rotundifolia*, *A. thaliana*, *V. vinifera*, *V. rotundifolia*, *V. amurensis*, *A. grossedentata*, and *S. lycopersicum* to create an upset plot (**Figure 2b**). The plot indicated that *A. grossedentata* possesses 193 unique gene families (comprising 1,075 genes) relative to other species. Enrichment analyses demonstrated that these unique gene families are predominantly involved in “metabolism” and “transport” pathways (**Supplementary Figure S3**).

Based on 175 single-copy homologous genes from 14 species, we constructed a high-confidence phylogenetic tree and estimated divergence times using the Bayesian relaxation molecular clock method (**Figure 2c**). Among these 14 species, *A. grossedentata* is most closely related to *C. rotundifolia*, diverging approximately 49.0 Mya. The lineage of *A. grossedentata* and the genus *Vitis* (*V. vinifera*, *V. rotundifolia*, *V. amurensis*) diverged from a common ancestor around 35.0 Mya (**Figure 2c**).

The expansion and contraction of gene families are pivotal in developing plant-specific traits and phenotypic diversity. Expanded gene families may acquire new functions, enhancing environmental adaptability. Analysis revealed that the MRCA (most recent common ancestor) had 27,183 gene families (**Figure 2c**). Compared to its closest ancestor, *A. grossedentata* significantly expanded 186 gene families (including 1,097 genes) and contracted 47 gene families (including 127 genes) (**Figure 2c**). GO enrichment analysis indicated that contracted gene families were enriched in “response to stimulus” and “methylation” (**Supplementary Figure S4a**), while expanded families were enriched in “secondary metabolite biosynthetic process” and “secondary metabolic process” pathways (**Supplementary Figure S4b**).

### 3.5 Whole-genome duplications of *A. grossedentata*

**Figure 3:**
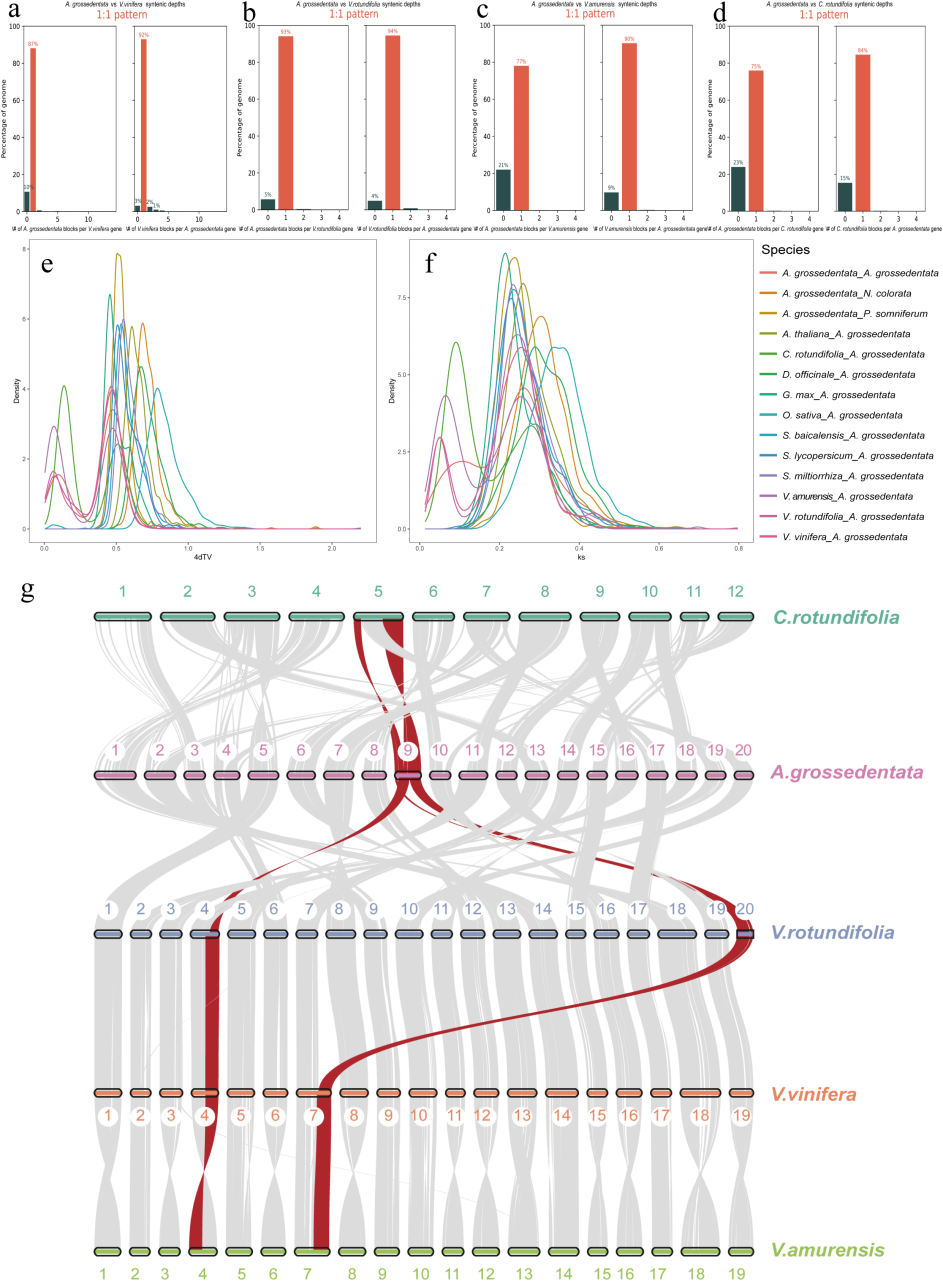
Comparative genomic and evolutionary analysis of *A. grossedentata*. **(a–d)** Syntenic depth between the representative Vitaceae species (*C. rotundifolia*, *V. vinifera*, *V. rotundifolia*, and *V. amurensis*) and *A. grossedentata*. **(e, f)** Distribution of 4dtv and *Ks* in 14 species. The dotted lines indicate the peaks of the *A. grossedentata* 4dtv and *Ks*. 4dTv, four-fold synonymous third-codon transversion position; *Ks*, synonymous substitution. **(g)** Synteny between *C. rotundifolia*, *V. rotundifolia*, *V. vinifera*, *V. amurensis* and *A. grossedentata*. The gray lines represent collinear blocks among species, while the red lines denote chromosomal fragment translocations between *A. grossedentata* and *C. rotundifolia*, V*. rotundifolia*, *V. vinifera*, and *V. amurensis*.

The 1:1 ratio of syntenic depth between the representative Vitaceae species (*C. rotundifolia*, *V. vinifera*, *V. rotundifolia*, and *V. amurensis*) and *A. grossedentata*, coupled with conserved syntenic patterns, indicates that both lineages experienced both recent and ancient whole-genome duplication (WGD) events (**Figures 3a–d**). Application of Tree2GD to the 14 genomes revealed 2 polyploidization events in the ancestor of ​*Vitales* Juss. ex Bercht. & J. Presl (11,681GDs) and that of *Rosales* Bercht. & J. Presl (15,800 GDs). And ratio of (AB)(AB) of the ancestor of ​*Vitales* (50.17%) and that of *Rosales* (51.14%) revealed strong phylogenomic signals for WGD. Tree2GD analysis was conducted on the genomes of 14 species, and it was found that *A. grossedentata* identified a total of 20,029 GDs (10.93%), indicating that *A. grossedentata* experienced two polyploid events (**Figure 2c**).

The distributions of 4DTv and *Ks* revealed two distinct peaks, indicating the occurrence of two WGD events (**Figures 3e, f**). The most recent WGD event aligns with those in *C. rotundifolia*, *V. vinifera*, *V. rotundifolia*, and *V. amurensis*, indicating a common WGD event in Vitaceae species. The other WGD event corresponds to the whole-genome triplication (WGT, γ event) common to core eudicots (Jaillon et al., 2007).

To understanding the chromosome evolution and phylogenetic relationships among Vitaceae species, we conducted genomic collinearity analysis of *C. rotundifolia*, *V. vinifera*, *V. rotundifolia*, *V. amurensis*, and *A. grossedentata* (**Figure 3g**). We observed fewer scattered dots in the comparison between *A. grossedentata* and *C. rotundifolia*, indicating a closer phylogenetic relationship between these two species compared to others (**Figure 3g**). Additionally, recombination and gene fragment rearrangement events were detected on chromosome 9 in *A. grossedentata*, including inversions and translocations (**Figure 3g**), which may have led to the high content of flavonoid compounds in *A. grossedentata*. Overall, these findings provide new insights into the chromosome evolution of *A. grossedentata* and will offer scientific evidence beyond the genus for studying important agronomic traits in *Vitis* species.

### 3.6 Integrated metabolome and transcriptome analyses

**Figure 4:**
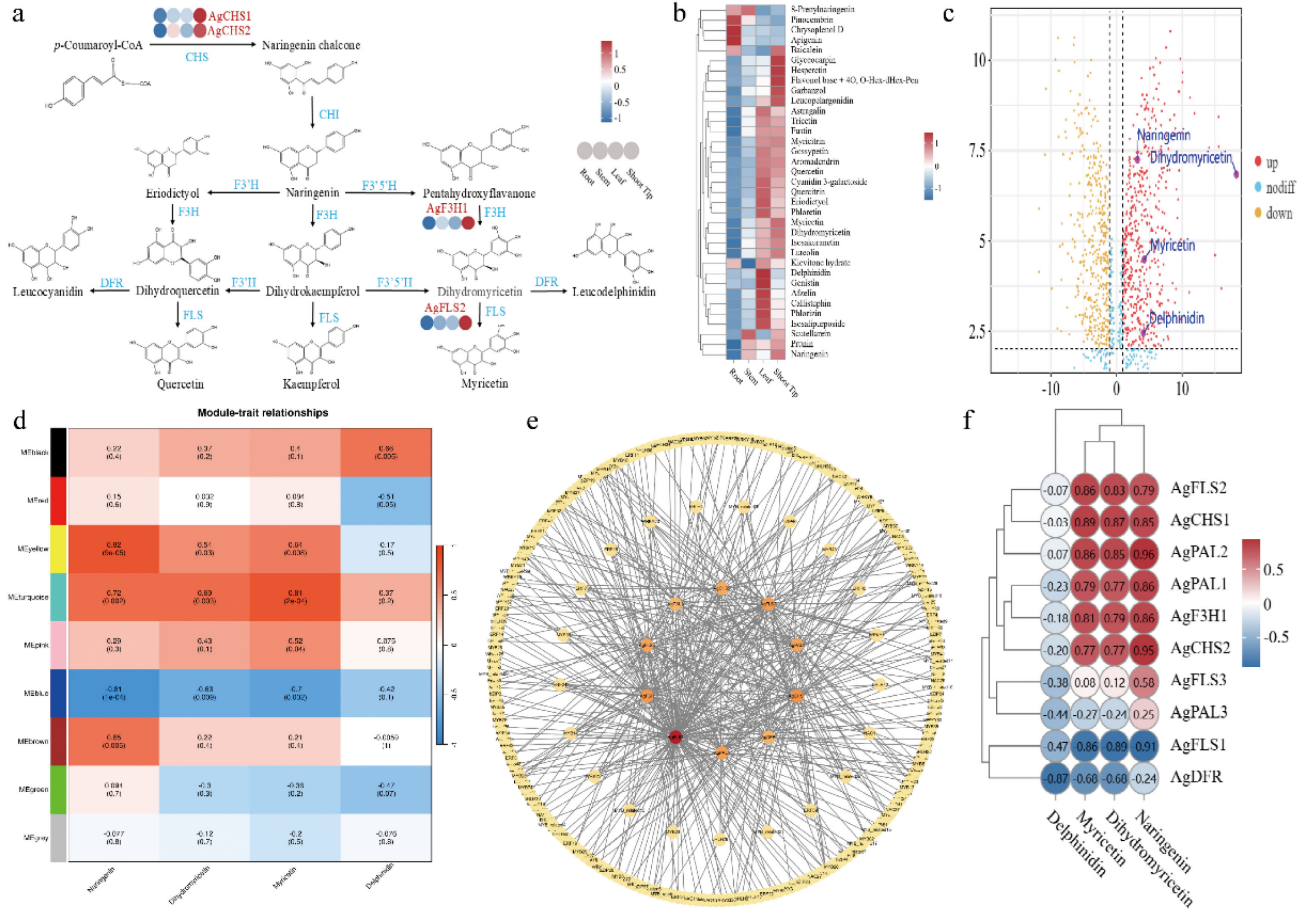
Metabolite levels, gene expression clustering, and flavonoid biosynthesis pathway. **(a)** Simplified flavonoid biosynthetic pathways in *A. grossedentata*. Enzymes in blue typeface; gene expression in rounded boxes. **(b)** Heat map showing expression of 35 metabolites in the flavonoid synthesis pathway across different tissues at identical developmental stages. Normalized (z-score) FPKM values color-coded by expression level. **(c)** Volcano plot of differential metabolites in roots and buds. x-axis: Log2 fold change; y-axis: -log10 P-value. Dot size represents VIP value; red: upregulated, yellow: downregulated, blue: non-significant. Labeled metabolites: naringenin, dihydromyricetin, myricetin, delphinidin. **(d)** A heatmap displaying the correlations between gene expression modules and flavonoid metabolites shows that rows represent distinct modules (ME), and columns denote flavonoid compounds. **(e)** Interaction networks of 10 key hub genes and 240 transcription factors in brown, yellow, blue, and turquoise modules mapped using Cytoscape. **(f)** Heat maps showing correlation between naringin, dihydromyricetin, myricetin, and delphinidin with 10 key genes identified by WGCNA.

Flavonoids are widely present in Vitaceae plants, including *A. grossedentata*, and play various roles in secondary metabolism. Previous research identified 138 flavonoid-related genes and isoforms, partially elucidating the flavonoid biosynthesis pathway (Li et al., 2020) (**Figure 4a**). We conducted metabolomics analyses on the roots, shoot tips, stems, and leaves of *A. grossedentata* to determine the correlation between flavonoids and gene expression. The reproducibility of the sequencing data was confirmed by PCA, OPLS-DA, and PLS-DA results for three biological duplicates (**Supplementary Figure S5**). Utilizing high-resolution LC-MS/MS analysis, 1,526 metabolites were detected across four tissues, including flavonoids (70), terpenoids (41), and carboxylic acids and their derivatives (37) (**Supplementary Figure S6**; **Supplementary Table S14**). Half of the 70 flavonoids (35) were abundant in the flavonoid biosynthesis pathway (Ko00941, **Supplementary Table S15**). These flavonoids were mainly expressed in shoot tips except for four metabolites found in roots (8-Prenylnaringenin, Pinocembrin, Chrysoplenol D, Apigenin) (**Figure 4b**). Earlier research indicated flavonoids synthesis mainly happens through dihydromyricetin accumulation, involving compounds like naringenin, dihydromyricetin, myricetin, and delphinidin (Yu et al., 2021). The differential expression metabolites in pairwise comparisons between the shoot tips and roots were most enriched in the flavonoid biosynthesis pathway (**Supplementary Figure S7**), with dihydromyricetin having the highest content in the shoot tips and showing the most significant differential expression between the shoot tips and roots (**Figure 4c**). Utilizing the near-complete of *A. grossedentata*, we performed transcriptome sequencing on the roots, stems, leaves, and shoot tips of PZY009 to identify key genes and transcription factors (TFs) involved in dihydromyricetin synthesis. The results of PCA for three biological replicates indicate high reproducibility of the sequencing data (**Supplementary Figure S8**). Our gene expression analysis discovered 10,566 genes (5,993 upregulated and 4,573 downregulated) when comparing shoot tips and roots (**Supplementary Table S16**; **Supplementary Figure S9**). GO and KEGG enrichment annotations revealed these genes’ functions were chiefly enriched in metabolic and secondary metabolite biosynthesis pathways, particularly flavonoid biosynthesis and isoflavonoid biosynthesis (**Supplementary Figure S10**).

### 3.7 Screening of key genes in the flavonoid compound biosynthesis pathway

Of 35 metabolites enriched in the flavonoid synthesis pathway (Ko00941), four key metabolites were identified: naringenin, dihydromyricetin, myricetin, and delphinidin. Therefore, we conducted WGCNA analysis using naringenin, dihydromyricetin, myricetin, and delphinidin with transcriptome data. Through WGCNA analysis, 17,085 genes from 12 samples were clustered into 9 modules (**Figure 4d**; **Supplementary Figure S11**; **Supplementary Table S17**). We focused on the yellow module (2,707 genes), brown module (3,234 genes), turquoise module (4,263 genes), and blue module (4,012 genes) (**Figure 4d**; **Supplementary Table S17**). Correlation analysis between genes and transcription factors in each module, using correlation coefficients ≥0.9 or ≤-0.9, identified 10 key enzyme genes and 364 transcription factors in the flavonoid biosynthesis pathway: yellow module (6 genes, 64 transcription factors), brown module (2 genes, 99 transcription factors), turquoise module (1 gene, 77 transcription factors), and blue module (1 gene, 124 transcription factors) (**Supplementary Tables S18, S19**). Tissue-specific expression profiling of *A. grossedentata* revealed pronounced spatial expression heterogeneity among ten screened key genes. Notably, the *AgF3H1* gene exhibited strong tissue-specific expression characteristics in shoot tips, demonstrating significantly higher transcriptional levels (FPKM values) compared to other examined tissues (P<0.05), with statistically significant differences. Systematic analysis of key structural genes in secondary metabolic pathways further uncovered marked tissue expression preferences: *AgFLS1* and *AgDFR* genes displayed high-abundance expression patterns in roots, while *AgPAL3* and *AgFLS3* genes showed significant tissue-specific overexpression in stems (**Supplementary Figure S12**). These tissue-specific expression patterns were subsequently validated through qRT-PCR, with experimental data demonstrating high consistency with transcriptomic analysis results (**Supplementary Figure S13**). Then, inter-group correlation analysis was performed on the 10 genes and 364 TFs, and 240 TFs were screened using correlation coefficients ≥0.95 or ≤-0.95 (**Supplementary Table S18**). The 10 genes and 240 TFs were used to calculate degree values using Cytoscape, and 21 TFs with degree values ≥5 were selected (**Figure 4e**; **Supplementary Table S19**). *A. grossedentata* is the plant with the highest flavonoid content, and the variation trend of dihydromyricetin basically reflects the changes in total flavonoid content. The inter-group correlation heatmap of 10 key genes with naringenin, dihydromyricetin, myricetin, and delphinidin showed that *AgF3H1*, *AgFLS2*, *AgCHS1*, *AgCHS2*, *AgPAL1*, and *AgPAL2* had the highest correlation with naringenin, dihydromyricetin, and myricetin (**Figure 4f**). In *A. grossedentata*, dihydromyricetin is synthesized by the *AgF3’5’H* enzyme gene (catalyzing dihydroquercetin or dihydrokaempferol) and the *AgF3H* enzyme gene (catalyzing pentahydroxyflayanone) (**Figure 4a**). Through WGCNA analysis, a *AgF3H1* gene was screened out, so we speculate that *AgF3H1* is the key gene for synthesizing dihydromyricetin.

### 3.8 Molecular docking of *AgF3H* genes

**Figure 5:**
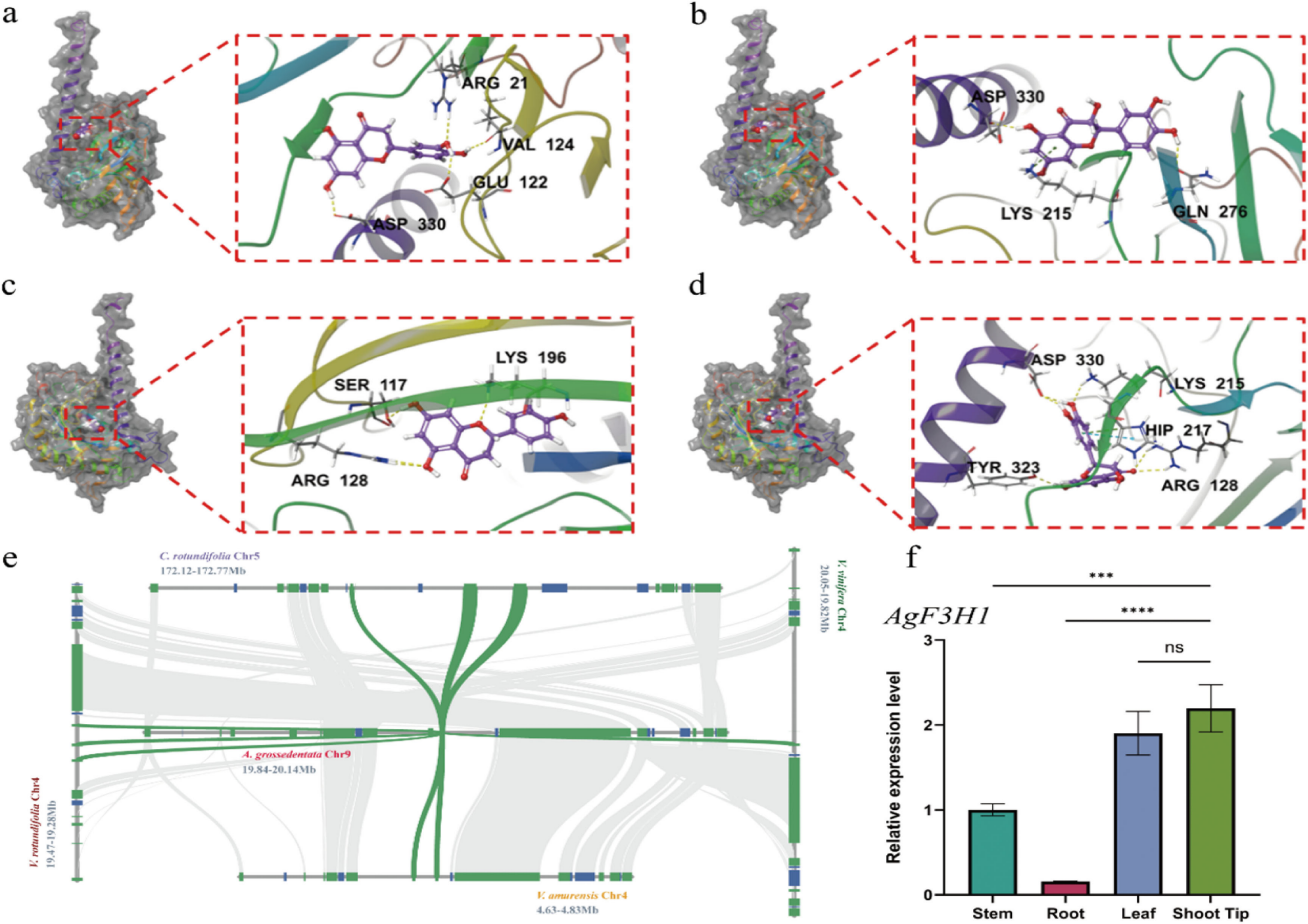
Bioinformatics analysis of *AgF3H1* and *AgF3H2* genes in *A. grossedentata*. **(a)** Molecular docking of *AgF3H1* with Eriodictyol. **(b)** Molecular docking of *AgF3H1* with pentahydroxyflavanone. **(c)** Molecular docking of *AgF3H2* with naringenin. **(d)** Molecular docking of *AgF3H2* with pentahydroxyflavanone. Yellow: hydrogen bonds; green: *π*-cation bonds; blue: *π*-*π* bonds. **(e)** Local collinearity analysis of chromosome 9 in *A. grossedentata* with corresponding chromosomes in *V.vinifera*, *V.amurensis*, *V.rotundifolia*, *C.rotundifolia* and *C.sinensis*. Green: homology of *AgF3H1*. **(f)** Quantitative RT-PCR analysis of *AgF3H1* expression across various tissues of *A. grossedentata*. Data are presented as mean ± SD from three independent replicates. Statistical significance was assessed using Student’s t-test: ***P < 0.001, ****P < 0.0001; n.s. indicates non-significant differences.

The *F3H* enzyme belongs to the 2-oxoglutarate-dependent dioxygenase (2-ODD) family, featuring a conserved N-terminal region and a C-terminal similar to the 4-hydroxylase alpha subunit (Cheng et al., 2014; Kawai et al., 2014). Within the flavonoid biosynthesis pathway of *A. grossedentata*, the *AgF3H* gene catalyzes the conversion of eriodictyol, naringenin, and pentahydroxyflavanone into dihydroquercetin, dihydrokaempferol, and dihydromyricetin, respectively (**Figure 4a**). Analysis of the genome sequence of *A. grossedentata* revealed two *F3H* genes (*AgF3H1* and *AgF3H2*). Subsequently, molecular docking was performed using the amino acid sequences of *AgF3H1* and *AgF3H2* with eriodictyol, naringenin, and pentahydroxyflavanone. The XP docking and MM-GBSA analysis showed that eriodictyol and pentahydroxyflavanone had docking scores of -8.032 and -6.121 with *AgF3H1*, and MM-GBSA binding free energies of -32.96 and -41.49 kcal/mol, respectively, indicating stable binding (**Supplementary Table S20**). Eriodictyol deeply penetrates the *AgF3H1* active pocket, forming hydrophobic interactions with ALA123 and VAL124, and hydrogen bonds with VAL124, GLU122, ARG21, and ASP330 (**Figure 5a**). Pentahydroxyflavanone also penetrates the *AgF3H1* active pocket, forming hydrophobic interactions with ALA334 and LEU333, and hydrogen bonds with ASP330 and GLN276, along with a *π*-Cation bond with LYS215 (**Figure 5b**). For *AgF3H2*, pentahydroxyflavanone and naringenin had docking scores of -6.747 and -6.025, and MM-GBSA binding free energies of -35.60 and -30.49 kcal/mol, respectively, indicating stable binding. Naringenin deeply penetrates the *AgF3H2* active pocket, forming hydrophobic interactions with PHE319, TYR323, PRO220, and LEU214, and hydrogen bonds with LYS196, SER117, and ARG128 (**Figure 5c**). Pentahydroxyflavanone penetrates the *AgF3H2* active pocket, forming hydrophobic interactions with PRO220 and TYR323, hydrogen bonds with TYR323 and LYS215, and additional hydrogen bonds with ASP330 and ARG128, as well as a *π*-Cation bond and a *π*-*π* bond with HIP217 (**Figure 5d**). In summary, the *AgF3H1* and *AgF3H2* genes in *A. grossedentata* are more likely to bind to pentahydroxyflavanone, and after binding, *AgF3H1* has higher stability with pentahydroxyflavanone than *AgF3H2*. Therefore, combined with the WGCNA results, we speculate that the *AgF3H1* gene is the key enzyme gene catalyzing the synthesis of dihydromyricetin from pentahydroxyflavanone. *AgF3H1* is located on chromosome 9, and interspecies collinearity analysis shows that chromosome 9 of *A. grossedentata* underwent gene rearrangement during evolution (**Figure 3c**). Local collinearity results show that the *AgF3H1* gene of *A. grossedentata* corresponds to the *F3H* genes on chromosome 4 of *Vitis* species and chromosome 5 of *C. rotundifolia* (**Figure 5e**), which is consistent with the interspecies collinearity results (**Figure 3c**). Through real-time fluorescence quantitative experiments, it was found that the expression level of the *AgF3H1* gene in different tissues is consistent with the transcriptome data (**Figure 5f**). In conclusion, we speculate that *AgF3H1* is the key gene for dihydromyricetin biosynthesis in *A. grossedentata*.

## 4 Discussion

The exclusive Chinese species, *Ampelopsis grossedentata*, is characterized by young stems and leaves rich in flavonoids, which are commonly used in functional health beverages and folk medicines. In this research, we utilized Illumina and PacBio sequencing in conjunction with high-throughput Hi-C technology to assemble a near-complete reference genome for *A. grossedentata*. The scaffold N50 is 30.45 Mb, and the contig N50 is 21.93 Mb, significantly surpassing Vitaceae species such as *Tetrastigma hemsleyanum* (contig N50: 2.15 Mb, scaffold N50: 86 Mb) (Zhu et al., 2023) and the medicinal plant *Neolamarckia cadamba* (contig N50: 0.82 Mb, scaffold N50: 29.20 Mb) (Zhao et al., 2022). The revised genome size of *A. grossedentata* is 555.42 Mb, anchored to 20 pseudochromosomes, marking the first near-complete reference genome of *Ampelopsis*. Its genome size is comparable to other Vitaceae species such as *V. amurensis* Rupr. (approximately 522.28 Mb) (Wang et al., 2024), *V. amurensis* (604.56 Mb) (Wang et al., 2021), *V. vinifera* (494.87 Mb) (Shi et al., 2023) and *V. rotundifolia* (413.91 Mb) (Huff et al., 2023). The *A. grossedentata* genome exhibits high heterozygosity (1.48%) and a large number of repetitive sequences (62.62%), which are higher than those in *Vitis* species such as *V. zhejiang-adstricta* (heterozygosity: 0.845%; repetitive sequences: 47.49%) (Li H. Y. et al., 2024), *V. amurensis* Rupr. (heterozygosity: 1.20%; repetitive sequences: 59.21%) (Wang et al., 2024), and *C. rotundifolia* (heterozygosity: 1.19%; repetitive sequences: 47.41%) (Xin et al., 2022). The short-read coverage reaches 99.29%, and the BUSCO evaluation shows that 98.80% of the genome is completely assembled, outperforming Vitaceae species *V. amurensis* Rupr. (BUSCO: 97.50%) (Wang et al., 2024), *V. amurensis* (reads: 98.58%; BUSCO: 94.60%) (Wang et al., 2021) and *V. vinifera* (BUSCO: 98.50%) (Shi et al., 2023). These findings indicate *A. grossedentata* genome is superior to other Vitaceae plants in sequence consistency, assembly accuracy and completeness, which provides a solid foundation for phylogenetic, gene function and molecular breeding research.

Throughout plant evolution, many species have experienced one or more ancient genome polyploidization events (Blanc and Wolfe, 2004, Jiao et al., 2011; Soltis and Soltis, 2016). Whole-genome duplication (WGD) has significantly contributed to plant speciation and the development of valuable traits (Rensing, 2014; Song et al., 2024). Due to their close relationship with the common ancestor of angiosperms, *Vitis* species and even Vitaceae plants are widely used in evolutionary analyses (Xin et al., 2022). Phylogenetic studies reveal that the divergence between *Cissus* and *Vitis* occurred approximately 49.0 million years ago (range: 31.4-69.1 million years ago), confirming previous estimates based on whole-genome data for the divergence of *C. rotundifolia* (68.41 million years ago, range: 44.1 to 89.8 million years ago) and *C. quadrangularis* (range: 60.19 to 84.68 million years ago) from *Vitis* (Xin et al., 2022; Li Q. Y. et al., 2024). However, there is also evidence suggesting that the divergence between *Cissus* and *Vitis* may have occurred around 38.07 million years ago (range: 21.38 to 67.28 million years ago) (Li et al., 2024). Therefore, the high-quality genome data of *A. grossedentata* provides strong support for resolving the evolutionary relationships and developmental positions among Vitaceae species. Ks and 4dtv show two peaks, indicating that *A. grossedentata* experienced the WGT-γ event common to angiosperms and a Vitaceae-specific whole-genome duplication event during its evolution (Jaillon et al., 2007; Tang et al., 2008). WGD events not only double the genome size but also facilitate the acquisition and loss of gene copies (Van de Peer et al., 2009). Previous studies have identified useful genes from WGDs associated with plant growth and metabolic pathways (Chae et al., 2014; Chakraborty, 2018). Collinearity analysis shows that the chromosome collinearity pattern among Vitaceae species is chaotic, and chromosome 9 of *A. grossedentata* has undergone chromosome reorganization and/or gene fragment rearrangement events during evolution. *A. grossedentata* expanded/contracted/lost a large number of genes in two WGD events, resulting in ancestral genes being scattered across multiple chromosomes. Gene family expansion has been recognized as a key driving factor in the formation of various plant species adapting to natural variations, and these expanded gene families increase plant adaptability to biotic and abiotic stresses (Renny-Byfield and Wendel, 2014). There are 1,114 expanded gene families in *A. grossedentata*, enriched in “secondary metabolite biosynthetic process” and “secondary metabolic process” pathways. In conclusion, we propose that after experiencing two WGD events and gene recombination events on chromosome 9, *A. grossedentata* accumulated key genes for synthesizing flavonoid compounds.

The mining of genetic resources and the screening of candidate genes for key traits enable researchers to identify crucial genetic variations and environmental adaptability. Through the integration of gene co-expression and flavonoid metabolomics analysis, we delineated the flavonoid biosynthetic pathway and its regulatory network in *A. grossedentata*. Dihydromyricetin is the most abundant flavonoid monomer compound in *A. grossedentata*, and its variation trend generally reflects the changes in total flavonoid content. In *A. grossedentata*, *AgF3H* and *AgF3’5’H* are key catalytic genes for synthesizing dihydromyricetin, which can catalyze pentahydroxyflavanone, dihydroquercetin, and dihydrokaempferol into dihydromyricetin. By analyzing the correlation between genes and metabolites using the WGCNA package, 10 key genes highly interconnected with flavonoid compound synthesis were screened in yellow, brown, blue, and turquoise modules. These genes include *AgPAL3*, *AgPAL2*, *AgPAL1*, *AgFLS3*, *AgFLS2*, *AgFLS1*, *AgF3H1*, *AgDFR*, *AgCHS2*, and *AgCHS1*. Transcriptome and qRT-PCR experiments showed that *AgF3H1* had the highest expression in shoot tips, followed by decreasing expression in leaves, stems, and roots, suggesting that high expression of the *AgF3H1* gene may lead to increased dihydromyricetin content in *A. grossedentata*, consistent with previous research results. Based on the near-complete genome sequence, we identified *AgF3H1* and *AgF3H2* in the *A. grossedentata* genome. Molecular docking showed that the *AgF3H* genes has a higher binding affinity with pentahydroxyflavanone, but *AgF3H1* (-41.49 kcal/mol) is more stable than *AgF3H2* (-30.49 kcal/mol) when bound to pentahydroxyflavanone. High expression of *F3H* can increase the flavonoid content in plant tissues. Studies have found that the *S. lycopersicum SlF3H* gene was transferred into *Nicotiana tabacum*, and the results showed that the flavonoid content in *N. tabacum* overexpressing the *SlF3H* gene was about 30% higher than in the wild type (Meng et al., 2015). The *CsF3Hs* gene from the tea plant was transferred into *A. thaliana*, and it was found that the content of most flavonol glycosides and oligomeric proanthocyanidins in the seeds significantly increased (20-40%), indicating that *CsF3Hs* plays a key role in flavonoid biosynthesis in *C. sinensis* (Han et al., 2017). Totally, the regulation of the *F3H* gene significantly impacts flavonoid metabolism and synthesis, underscoring the pivotal role of *AgF3H* in the biosynthesis of flavonoid compounds and derivatives in *A. grossedentata*. Therefore, combined with the WGCNA results, we speculate that the *AgF3H1* gene is a key enzyme gene catalyzing the synthesis of dihydromyricetin from pentahydroxyflavanone. In subsequent research phases, comprehensive functional validation of the *AgF3H1* gene will be performed through both homologous and heterologous systems. Systematic investigations will include overexpression analysis in native host species alongside heterologous expression in model organisms, complemented by targeted gene knockout experiments using CRISPR/Cas9-mediated genome editing. These multi-platform approaches will elucidate the gene’s regulatory mechanisms in flavonoid biosynthesis pathways and its pleiotropic effects on plant physiological processes. The resulting data will establish a theoretical foundation for molecular breeding programs aimed at enhancing phytochemical profiles in crops, while concurrently providing technical parameters for developing nutraceutical-enriched agricultural products through metabolic engineering strategies.

In summary, we present the first near-complete genome assembly of *A. grossedentata*, providing comprehensive genomic data crucial for in-depth studies of this species and other medicinal and edible plants. Comparative genomic evolutionary analysis sheds light on the evolutionary trajectory of Vitaceae. The discovery of candidate genes involved in flavonoid biosynthesis paves the way for future genetic enhancement of *A. grossedentata*.

## Data Availability

The genome assembly and raw sequencing data for Ampelopsis grossedentata have been submitted to NCBI under project ID PRJNA1117225.
