# Supplementary material for: The near-complete genome assembly of Ampelopsis grossedentata provides insights into its origin, evolution, and the regulation of flavonoid biosynthesis
Source: Front Plant Sci. 2025 Aug 11;16:1580779. doi: 10.3389/fpls.2025.1580779 (PMC12375659; doi:10.3389/fpls.2025.1580779)
Supplement: Supplementary file 1 [file DataSheet1.docx]

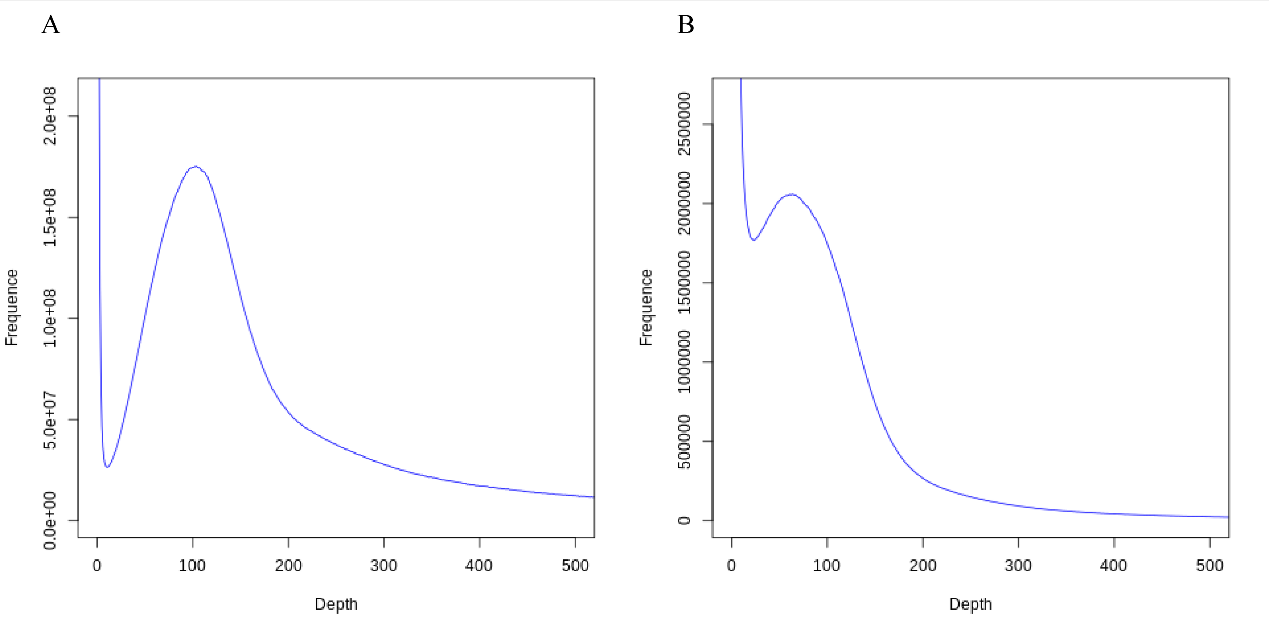


Figure S1. Genome survey of *A. grossedentata* using 17-mer analysis (Left: *K-mer*=17 Depth and *K-mer* count frequency distribution; Right: *K-mer*=17 Depth and *K-mer* species frequency distribution).


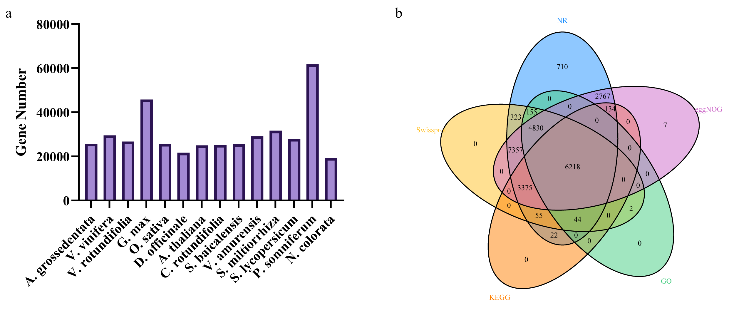


Figure S2. (a) Total number of predicted genes in 14 species. (b) Venn map of gene function annotation in different databases.**
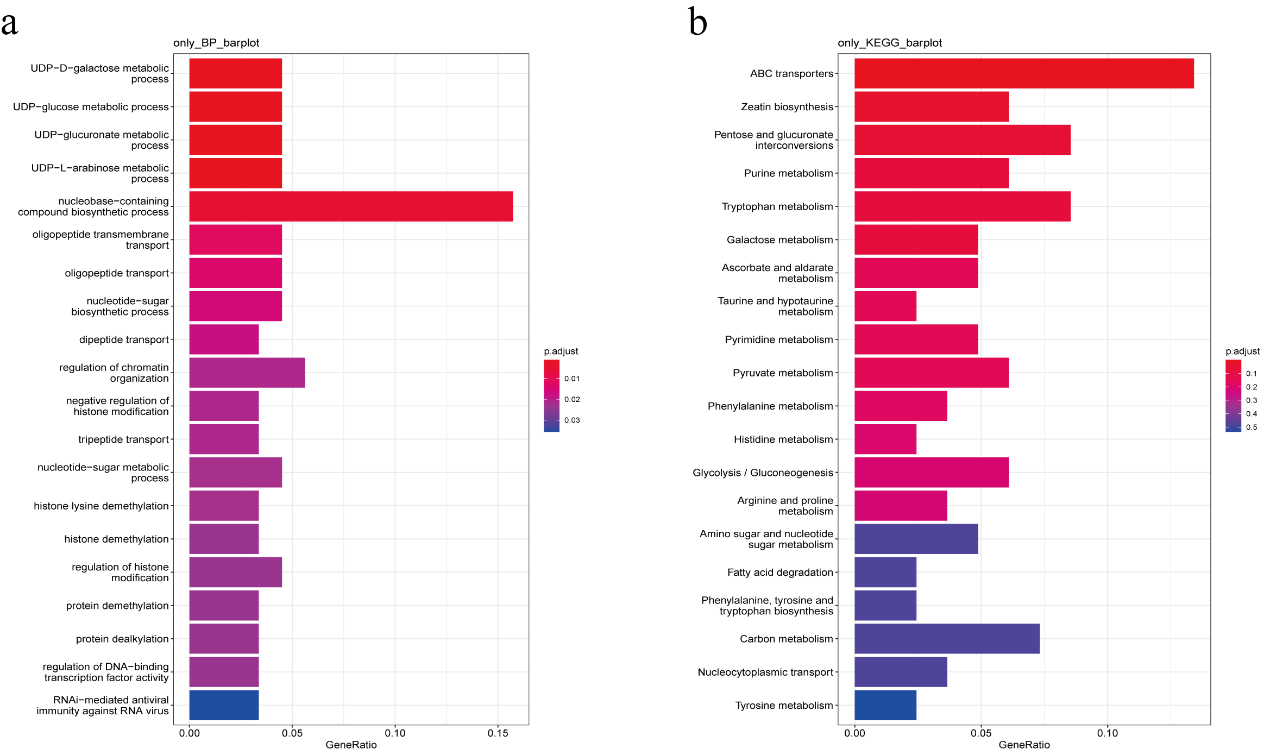
**

Figure S3. GO and KEGG enrichment analysis of *A. grossedentata*-specific gene families.
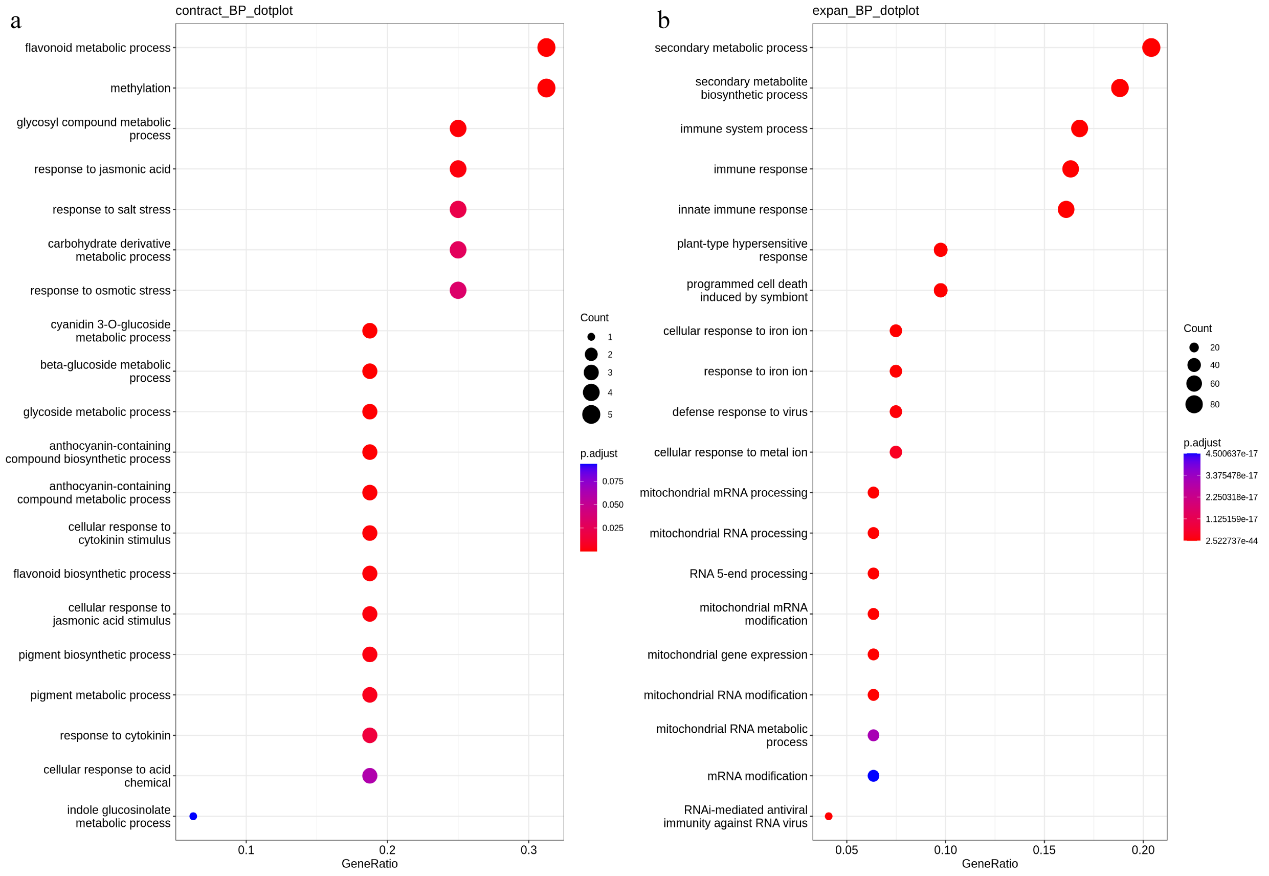


Figure S4. GO and KEGG enrichment analysis of expanded and contracted gene families during *A. grossedentata* evolution.


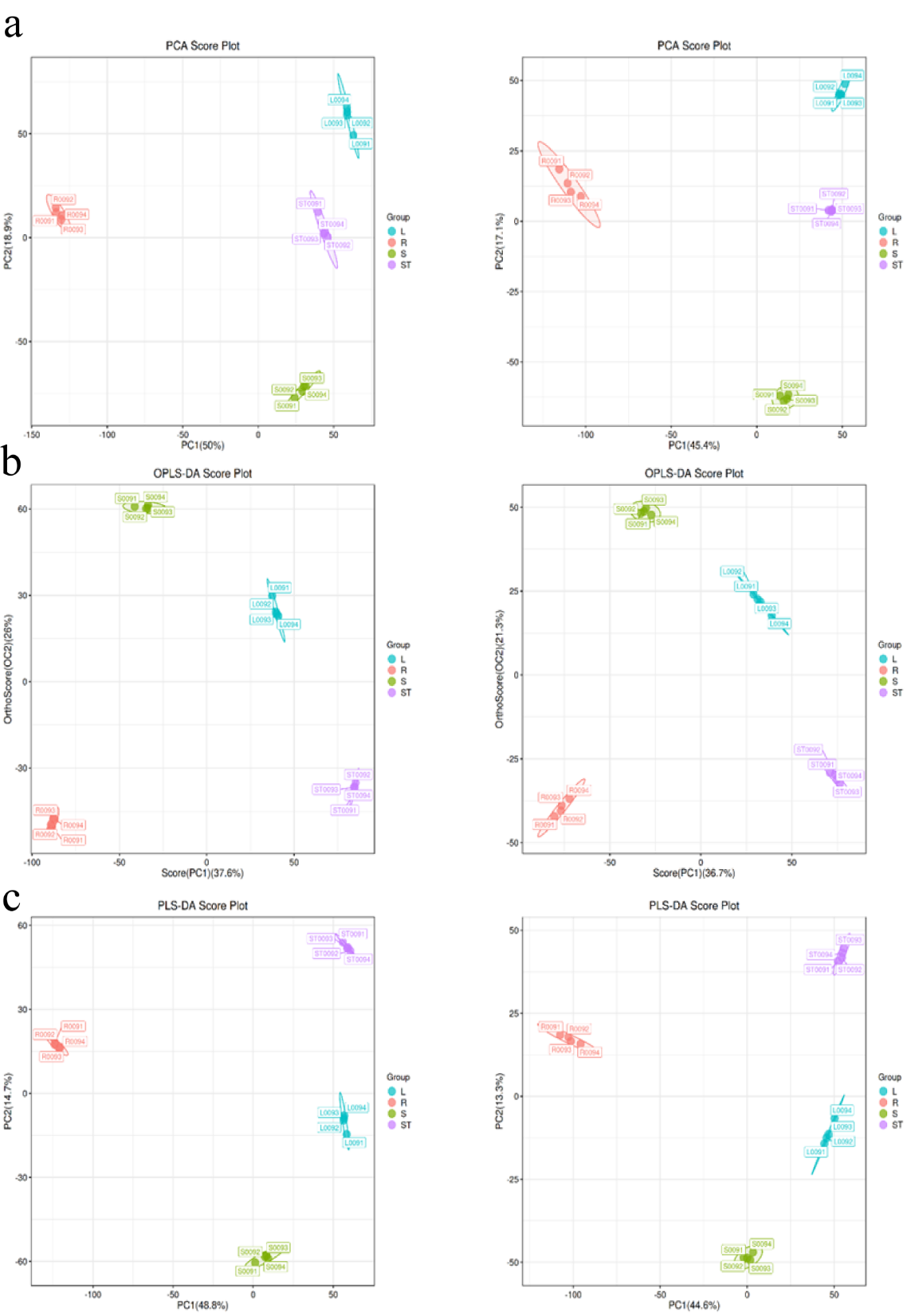


Figure S5. PCA analysis of metabolomics.
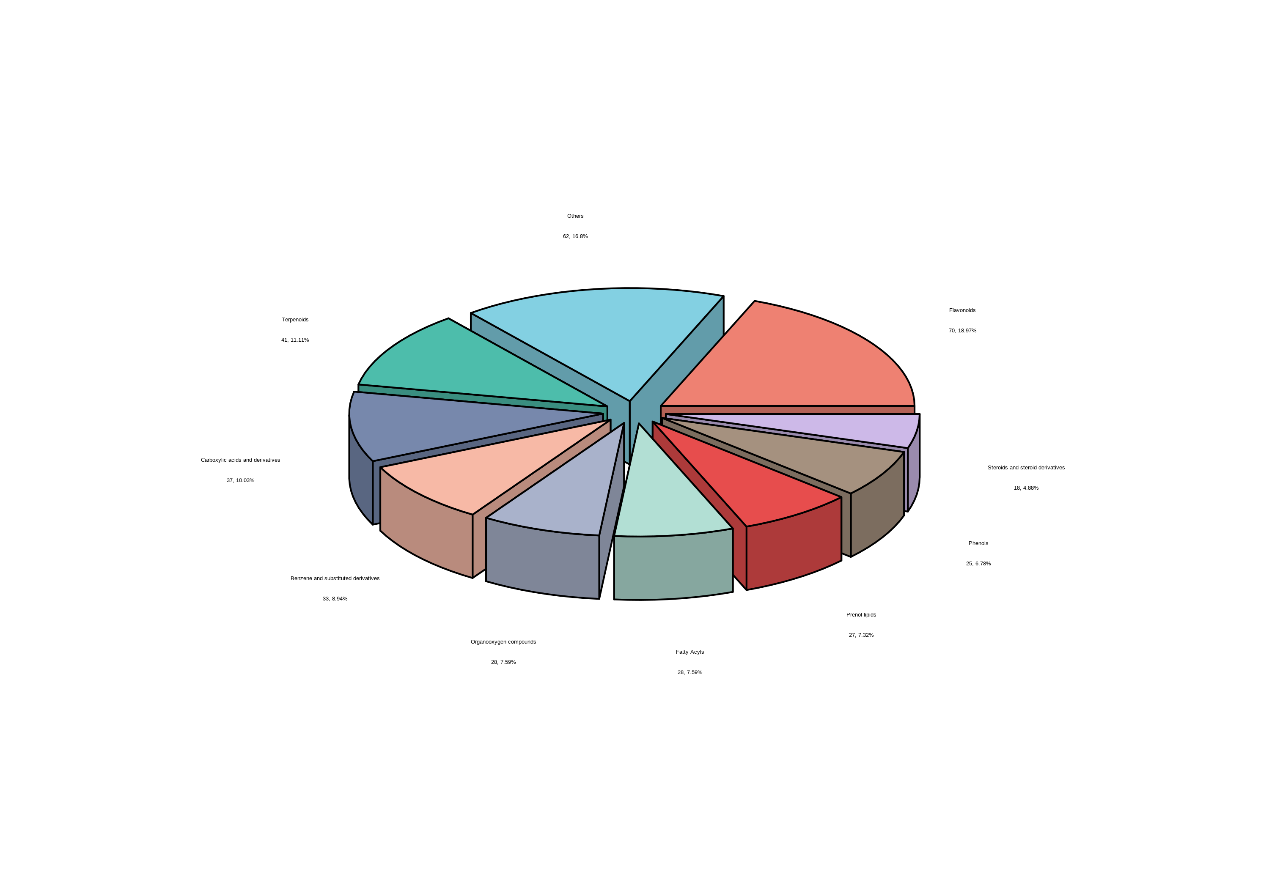


Figure S6. Pie chart of differential metabolite classification identified by metabolomics.
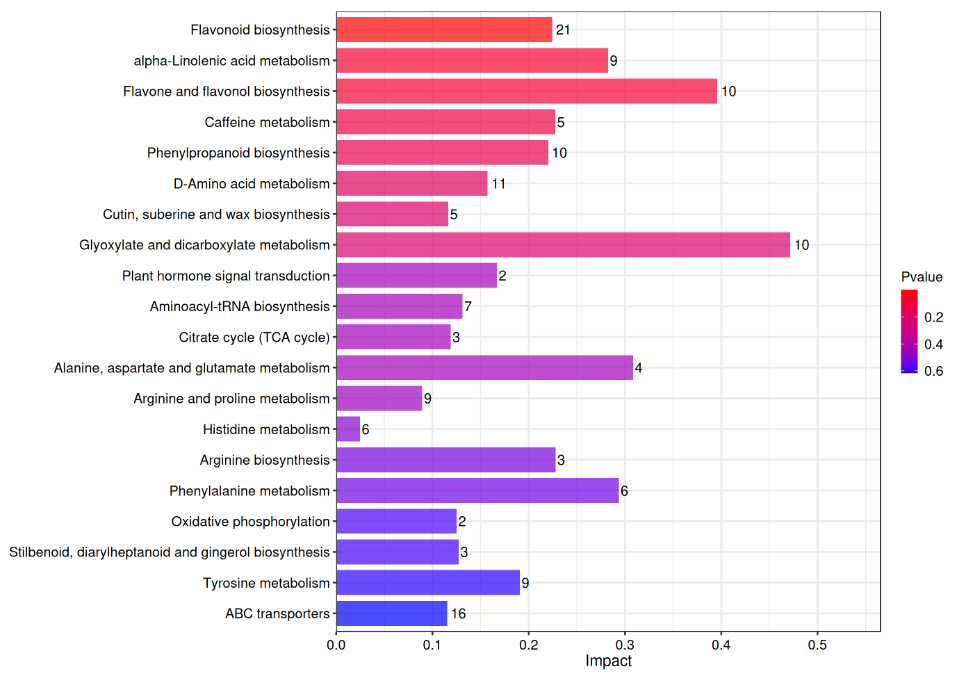


Figure S7. Enrichment results of differential metabolites.


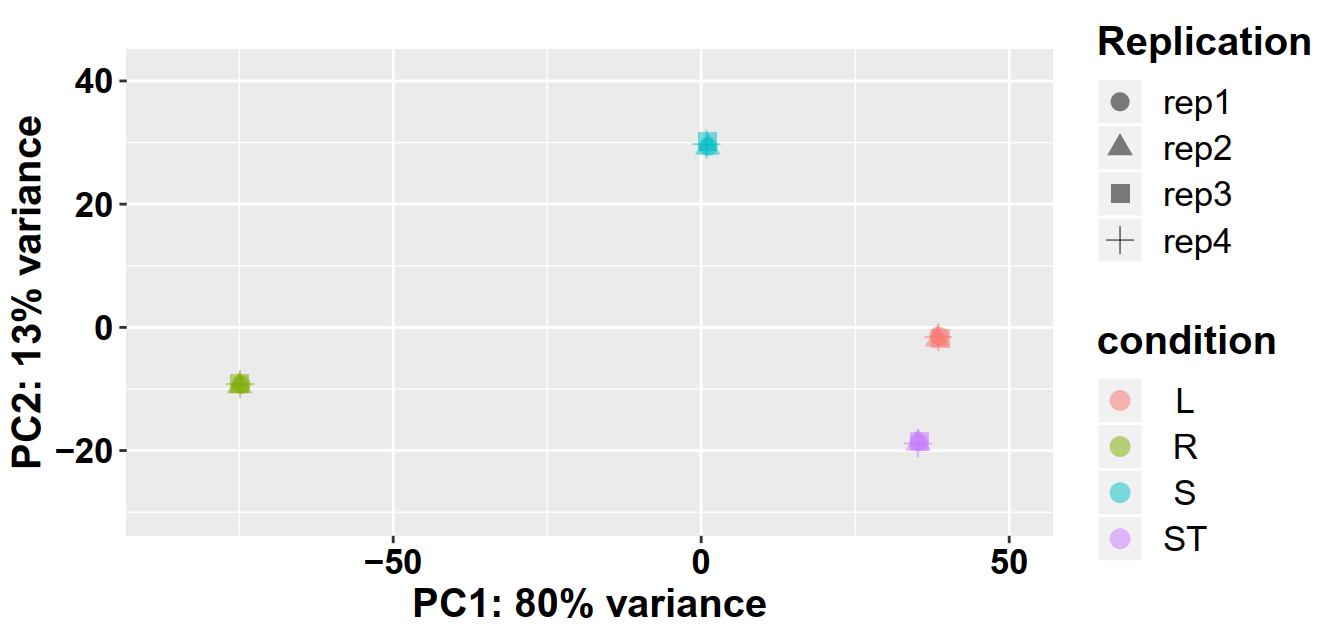


Figure S8. PCA analysis of transcriptome sequencing.


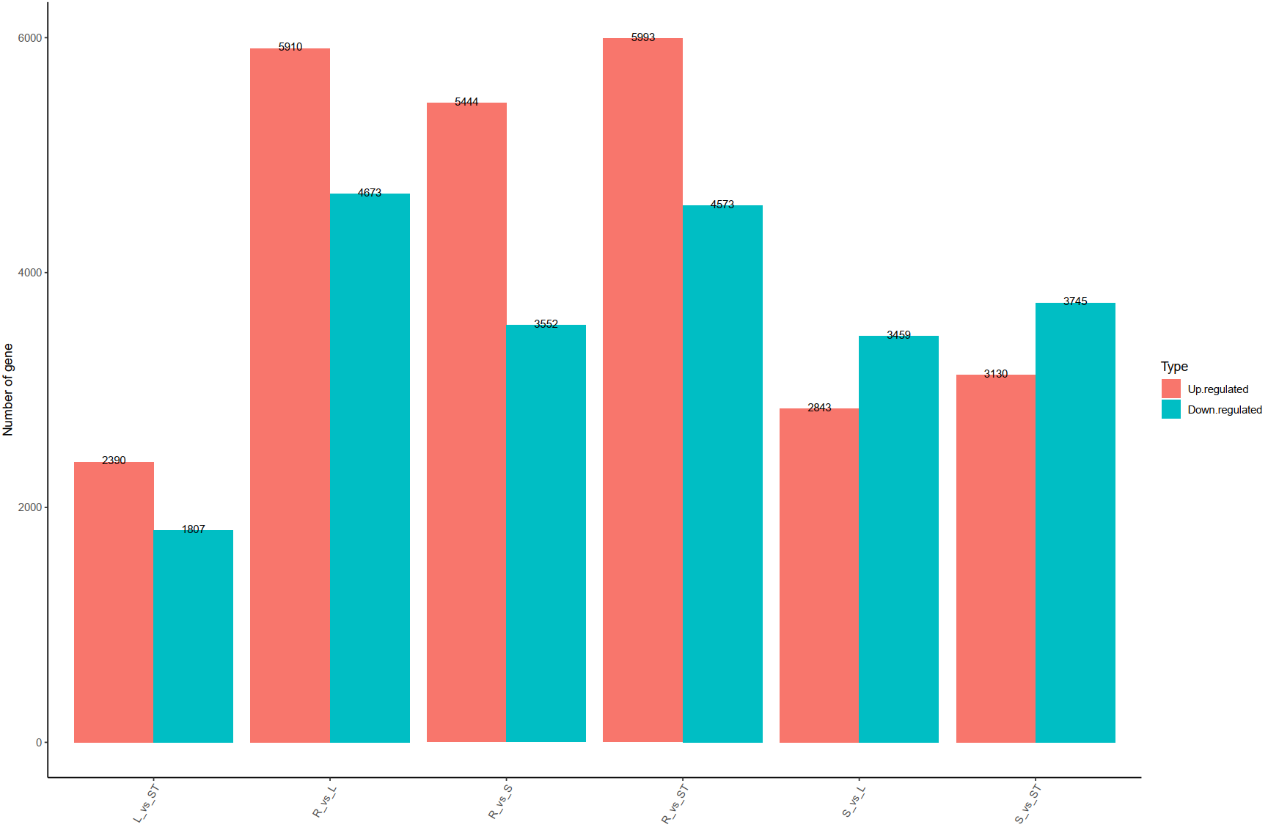


Figure S9. Comparison of differentially expressed genes between pairs of tissues in transcriptome sequencing.
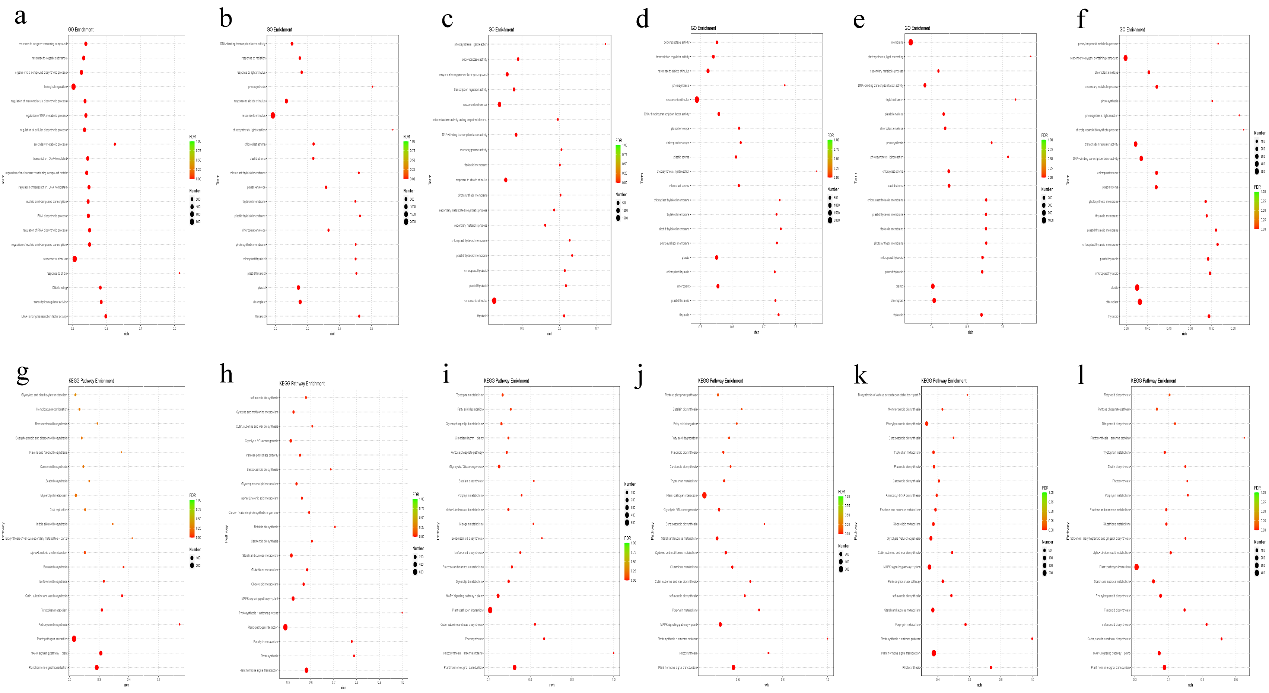


Figure S10. GO and KEGG enrichment analysis of differentially expressed genes between pairs of tissues in *A. grossedentata*.
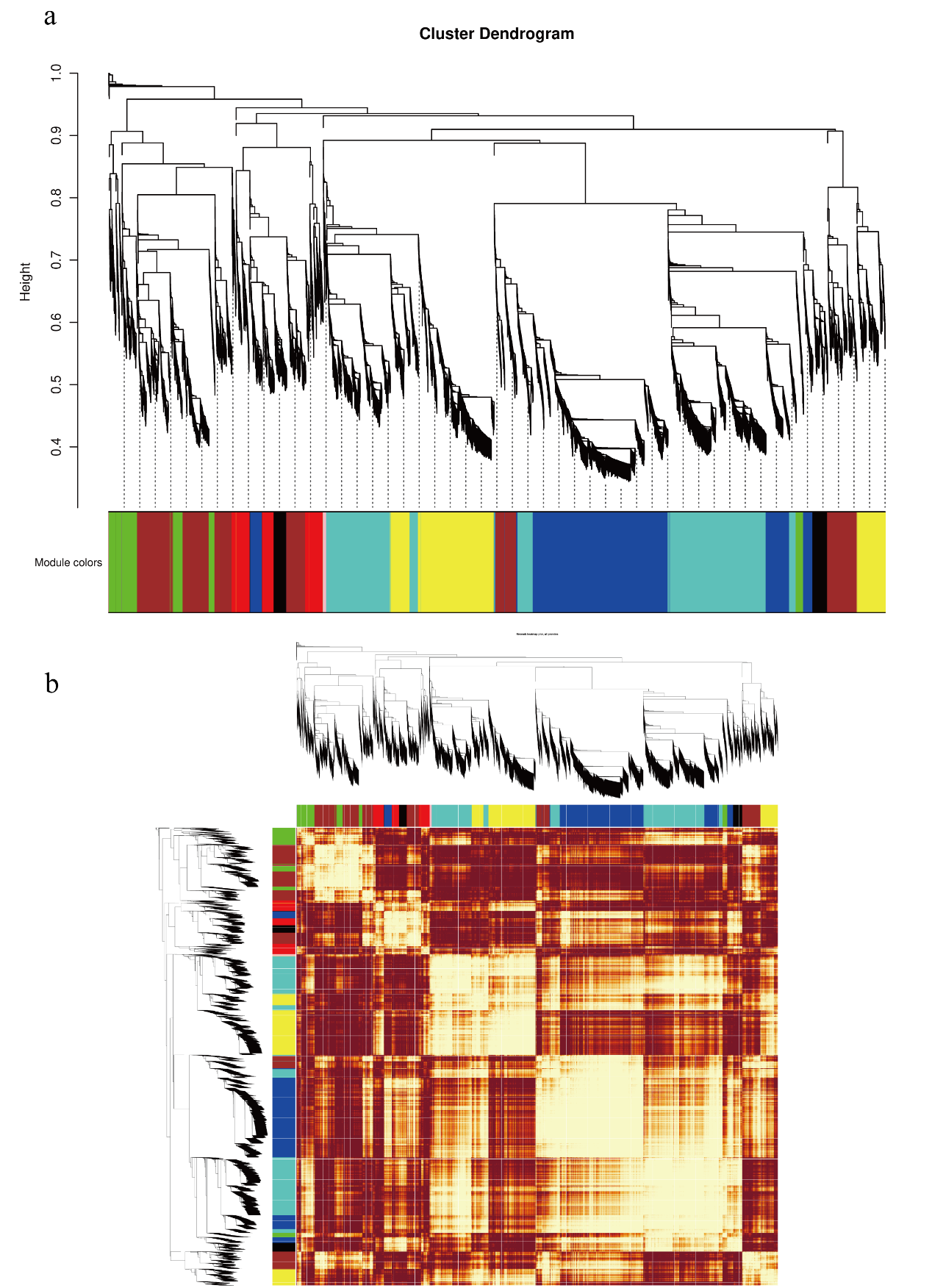


Figure S11. Cluster dendrogram showing 9 gene co-expression modules was built based on the dissimilarity of the topological overlap.


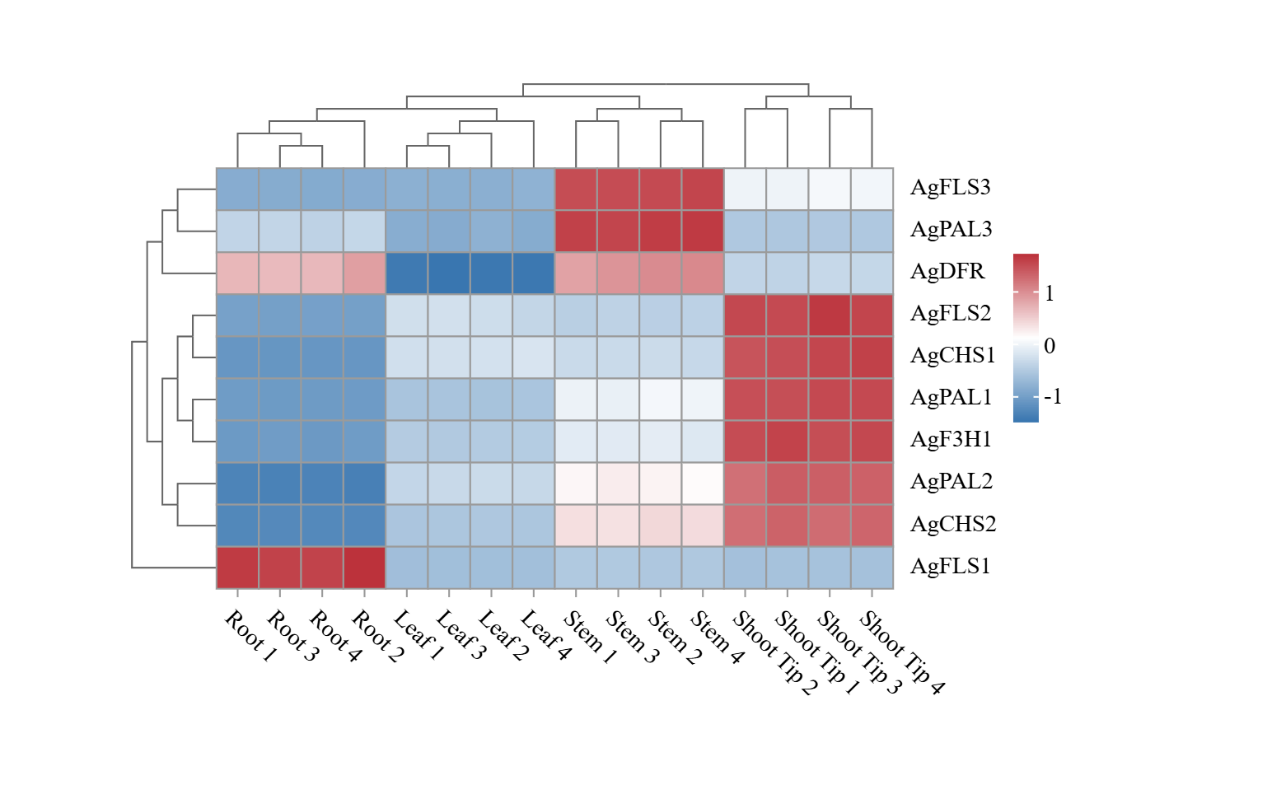


Figure S12. Expression heat maps of 10 key genes in different tissues.


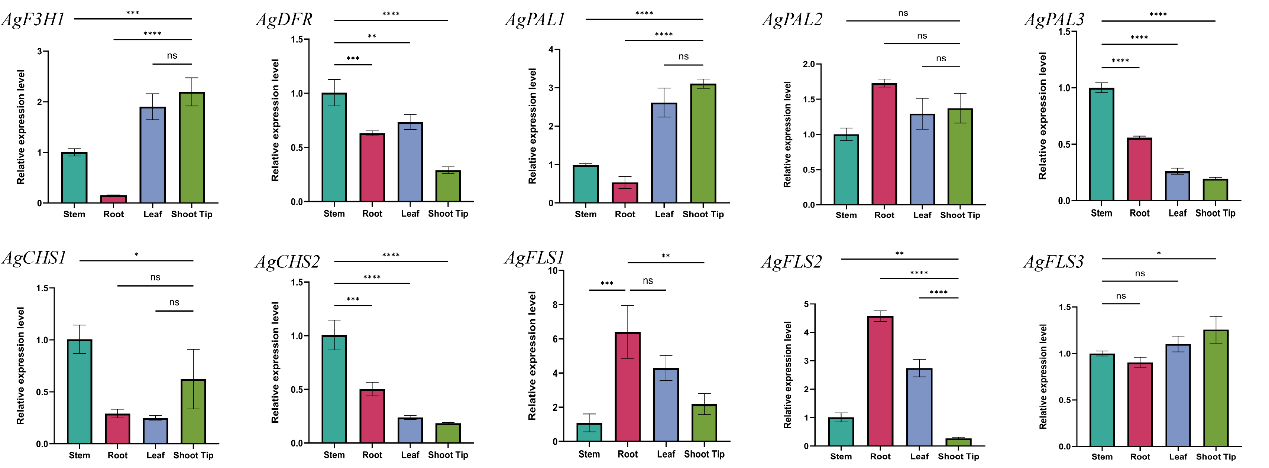


Figure S13. The qRT-PCR results of the 10 key genes screened out
